# Supplementary material for: Distinguishing faceted oxide nanocrystals with 17O solid-state NMR spectroscopy
Source: Nat Commun. 2017 Sep 18;8:581. doi: 10.1038/s41467-017-00603-7 (PMC5603560; doi:10.1038/s41467-017-00603-7)
Supplement: Supplementary file 1 — Supplementary Information [file 41467_2017_603_MOESM1_ESM.pdf]

Description of Supplementary Files

File Name: Supplementary Information
Description: Supplementary Methods, Supplementary Figures, Supplementary Tables, Supplementary Notes, and Supplementary References.

File Name: Peer Review File

Supplementary Methods

Preparation of NS001-TiO_2

In a typical experimental procedure, 25 mL of $Ti(OBu)_4$ and 3 mL of HF solution (40 wt%) were mixed in a 100 mL Teflon autoclave, and then kept at 180 °C for 24 h (***Caution***, *HF solution is extremely corrosive and it should be handled with extreme care!*). After being cooled to room temperature, the white powder was separated by high-speed centrifugation and washed with ethanol twice, with 0.1 M NaOH aqueous solution and distilled water repeatedly to remove residual fluorine species. At last, these products were dried in an electric oven under air flow at 80 °C for 6 h.

Preparation of NO101-TiO_2

First, $TiCl_4$/HCl aqueous solution was added to $NH_3{\cdot}H_2O$ aqueous to form $Ti(OH)_4$ precursor. In detail, 6.6 mL of $TiCl_4$ was added to 0.43 mol/L aqueous HCl drop by drop under strong stirring in an ice bath to obtain aqueous $TiCl_4$. This aqueous solution was then added to a 5.5 wt % aqueous $NH_3{\cdot}H_2O$ drop by drop under stirring, forming white $Ti(OH)_4$ precipitate. Afterward, the pH of the mixture was adjusted to 6~7 using 4 wt% $NH_3{\cdot}H_2O$ aqueous solution. After aging at room temperature for 2 h, the suspension was centrifuged, and the precipitate $Ti(OH)_4$ was washed by water twice and ethanol once.

The $Ti(OH)_4$ precursor (2.0 g) was dispersed in a mixture of 15 mL water and 15 mL isopropanol. After stirring and ultrasonic treatment, a suspension was obtained. The suspension was then transferred to a 50 mL Teflon-lined autoclave and heated at 180 °C for 15 h. After the reaction, the products were collected by centrifugation and washed with ethanol twice. Finally the product was washed with 0.1 M NaOH aqueous solution and distilled water repeatedly to remove chlorine.

Considering the fact that (101) facets are less reactive, the hydrothermal reaction time is shortened to 15 h, in order to obtain octahedral nanocrystals with a larger surface area, which facilitates the subsequent ^{17}O isotopic labeling.

Preparation of NF2-TiO_2

NF2-TiO_2 was prepared with a similar method used for NO101-TiO_2, but with a much shorter hydrothermal time, 1.5 h. This short hydrothermal time is insufficient for the formation of ordered surface structure, thus NF2-TiO_2 is a non-faceted anatase TiO_2 sample[1].

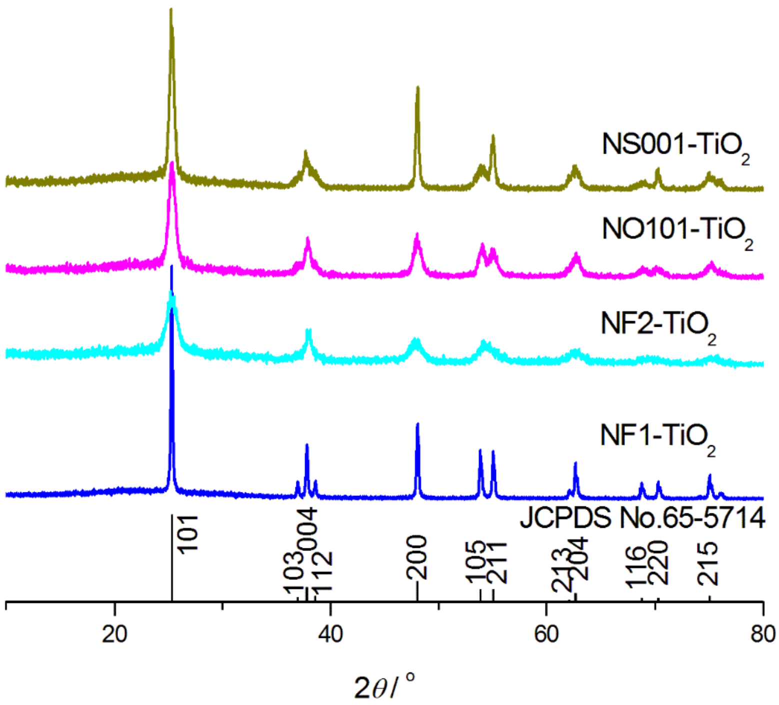

Supplementary Figure 1. XRD patterns of anatase TiO_2 samples. Faceted NS001-TiO_2 (dark yellow), NO101-TiO_2 (magenta), and non-faceted anatase TiO_2 nanoparticle samples NF2-TiO_2 (cyan) and NF1-TiO_2 (blue), as well as the PDF card of anatase TiO_2 (black) are presented.

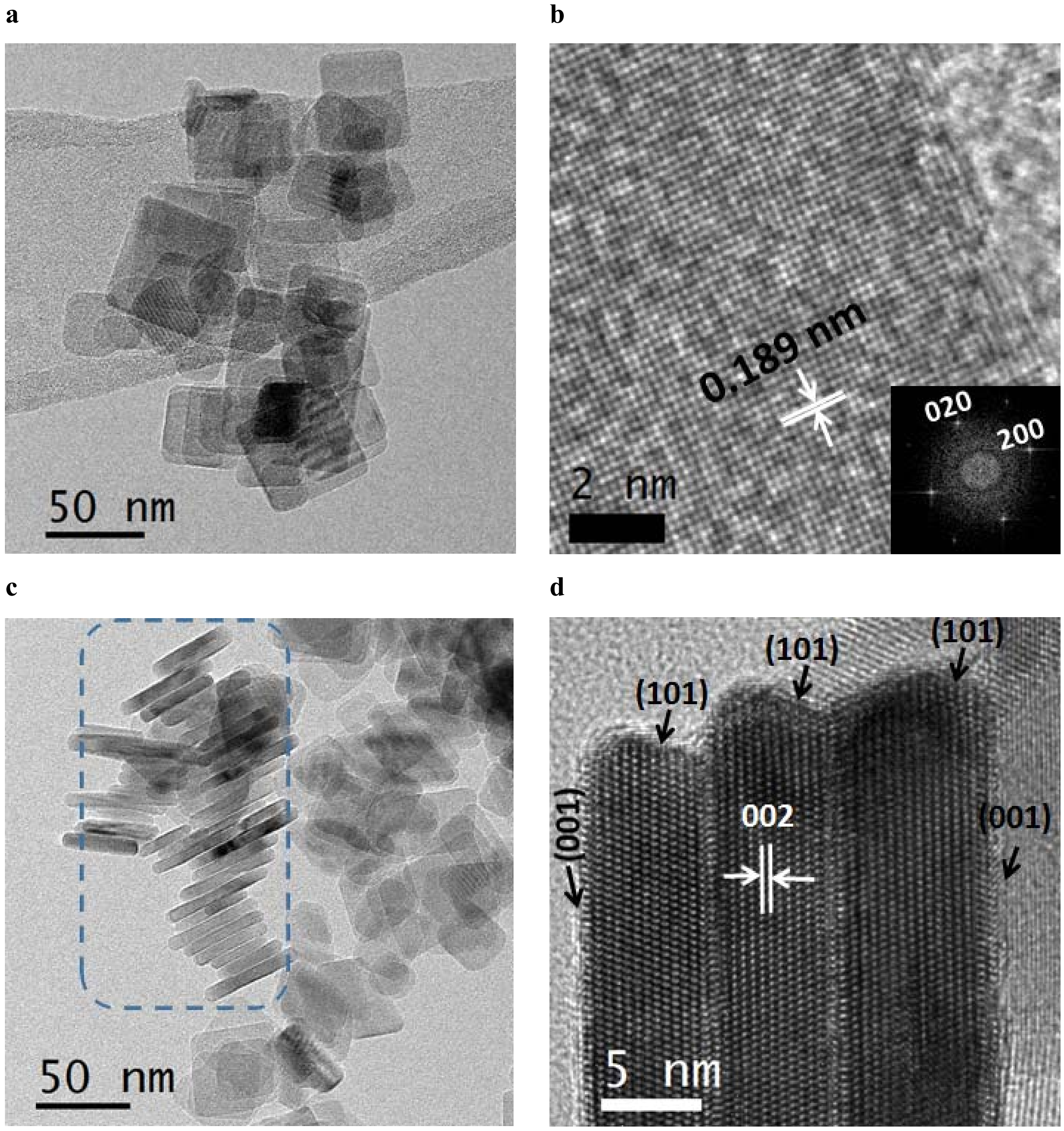

Supplementary Figure 2. HRTEM images of the as-prepared anatase TiO_2 nanosheets (NS001-TiO_2). Shape parameters of the nanosheets circled with dashed lines in **c** are surveyed in Supplementary Table 1 and interpreted in Supplementary Note 1.

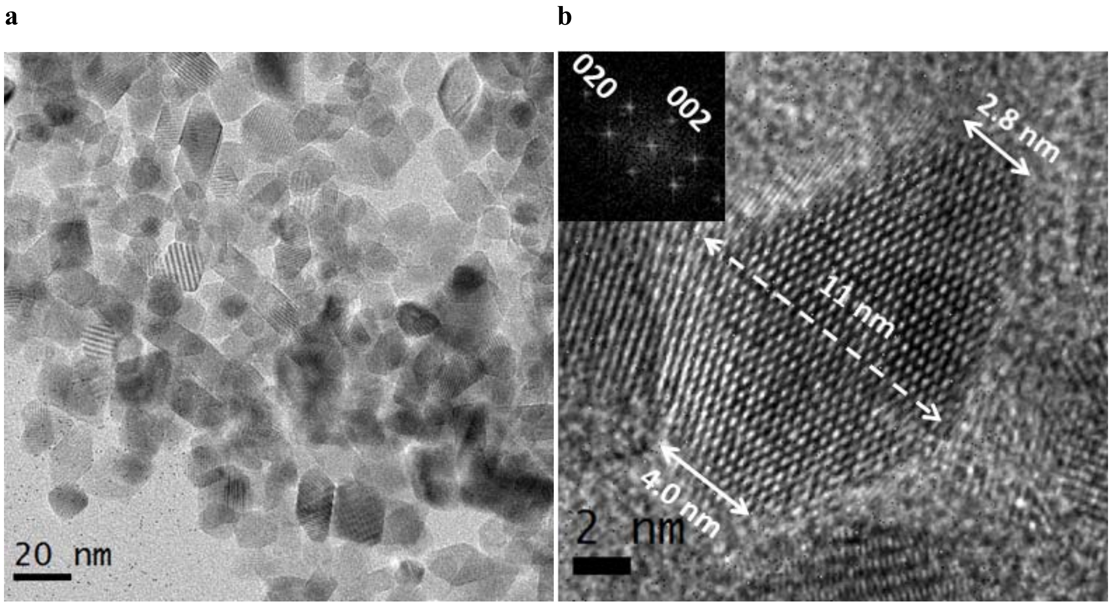

Supplementary Figure 3. HRTEM images of the anatase TiO_2 nano-octahedra (NO101-TiO_2). **a**, Low magnification and **b**, high resolution. Inset in **b** shows FFT image of the particle.

Supplementary Table 1. Shape parameters of the nanosheets circled in Supplementary Fig. 2c. A, B and P_{001} are interpreted in Supplementary Note 1.

***A*/nm**	***B*/nm**	***A*/*B***	**P_{001}**
32.5	30.0	1.08	0.680
38.0	35.5	1.07	0.717
30.0	28.0	1.07	0.714
37.0	33.5	1.10	0.635
26.5	25.0	1.06	0.749
30.0	28.0	1.07	0.714
34.0	32.0	1.06	0.741
41.0	39.0	1.05	0.779
21.0	19.0	1.11	0.625
27.5	25.5	1.08	0.694
20.5	19.5	1.05	0.779
44.0	43.0	1.02	0.887
55.5	54.5	1.02	0.909
44.5	42.5	1.05	0.793
35.0	33.5	1.04	0.802
38.0	36.0	1.06	0.764
39.5	38.0	1.04	0.821
29.0	28.0	1.04	0.836
36.0	34.0	1.06	0.753
32.0	30.0	1.07	0.729
28.5	26.5	1.08	0.702
22.0	21.0	1.05	0.791
26.0	24.0	1.08	0.680
37.0	35.0	1.06	0.759
	**Average**		0.766±0.056

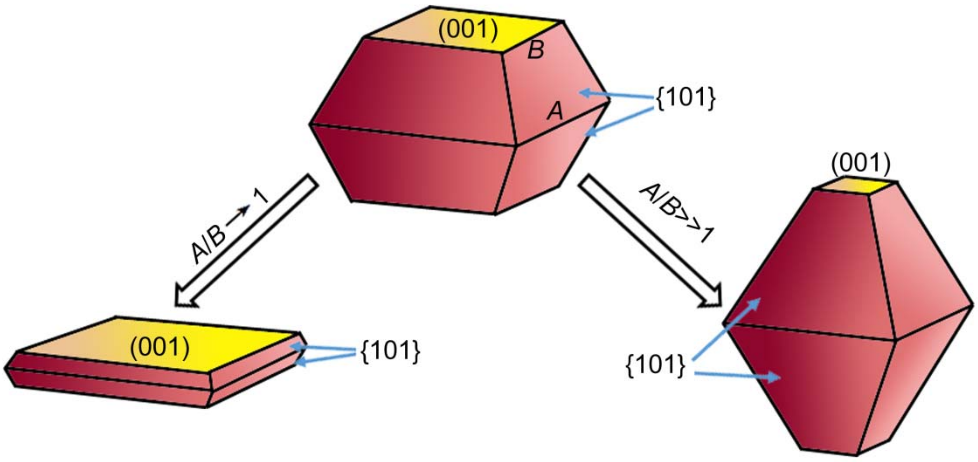

Supplementary Figure 4. Schematic diagram of anatase TiO_2 nanocrystals with {101} and {001} facets exposed.

Supplementary Note 1

As shown in Supplementary Fig. 4, an anatase TiO_2 nanocrystal can be described as a bipyramid with {001} truncation facets[2], where A denotes lengths of the side of the bipyramid, and B denotes the lengths of the side of the {001} square. When B is similar to A, it turns out to be a nano-sheet, with most of the surface being {001} facets. However, when B is much smaller than A, it turns out to be an octahedron, with most of the surface being {101} facets. The percentages of {001} and {101} surface can be estimated by the ratio of A/B, using P_{001}=cos68.3°/[$(A/B)^2$+cos68.3°-1], and P_{101}=1-P_{001}, respectively, according to previous reports[2,3].

For NS001-TiO_2, the two parameters (A and B) were surveyed by examining the circled nanosheets shown in Supplementary Fig. 2c. The data are shown in Supplementary Table 1, which gives a percent of 77% ± 6% of the exposed {001} facets.

For NO101-TiO_2, the ratio of A/B are estimated to be 3.3 ± 0.6 by measuring a representative nanoparticle, which is shown in Supplementary Fig. 3b. Therefore, the percent of exposed {101} surface is determined to be 96% ± 1%.

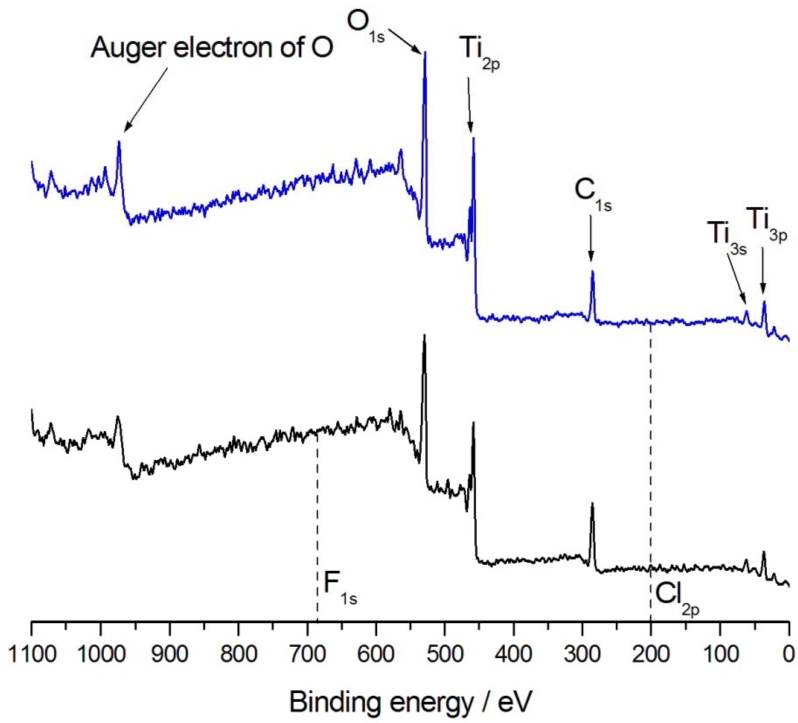

Supplementary Figure 5. XPS patterns of two faceted anatase TiO_2 samples. F^- (686 eV) or Cl^- (200 eV) species are not observed on surface of NS001-TiO_2 (bottom) and NO101-TiO_2 (top).

Supplementary Table 2. Properties of the samples.

Sample	Hydrothermal reaction time / h	BET surface area / $m^2 \cdot g^{-1}$	Crystal size* / nm	Content of C / wt %	Content of N / wt %
**NS001-TiO_2**	24	67	17.8	0.48	0.09
**NO101-TiO_2**	15	99	13.6	0.19	0.23
**NF1-TiO_2**	/	17	50.3		
**NF2-TiO_2**	1.5	259	9.2		

* Crystal size is given by analyzing the FWHM broadening of the (101) peak in XRD pattern.

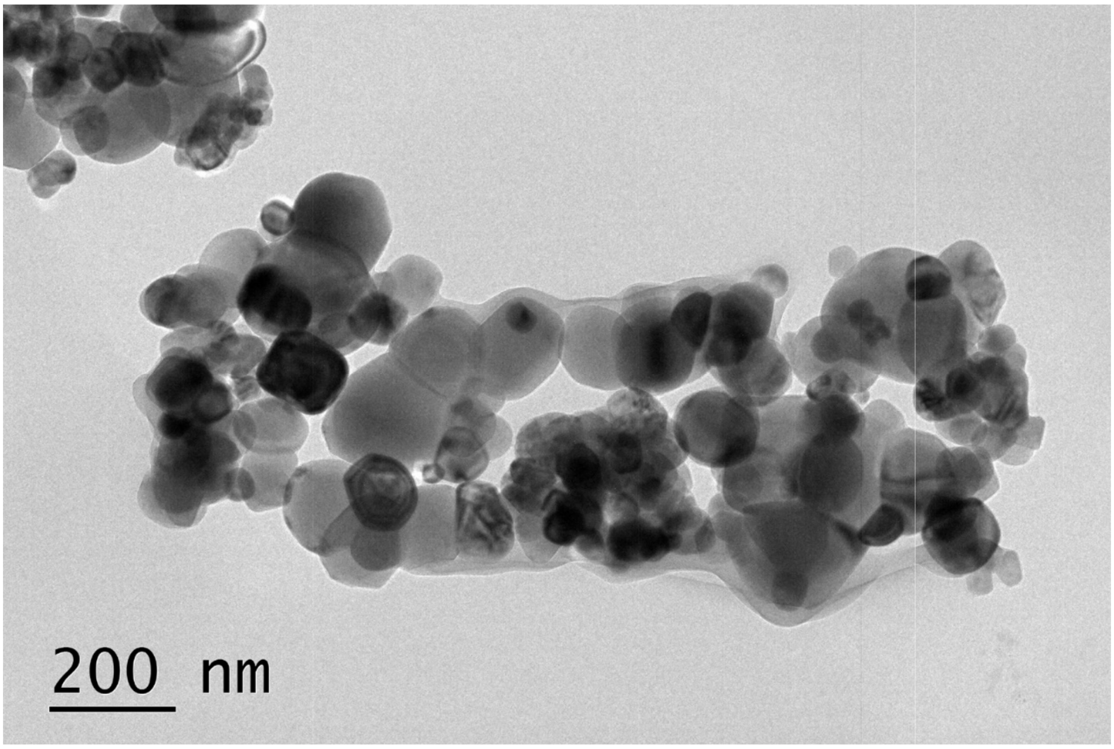

Supplementary Figure 6. TEM image of the non-faceted anatase TiO_2 sample, NF1-TiO_2. The size of the particles are about 50 ~ 200 nm.

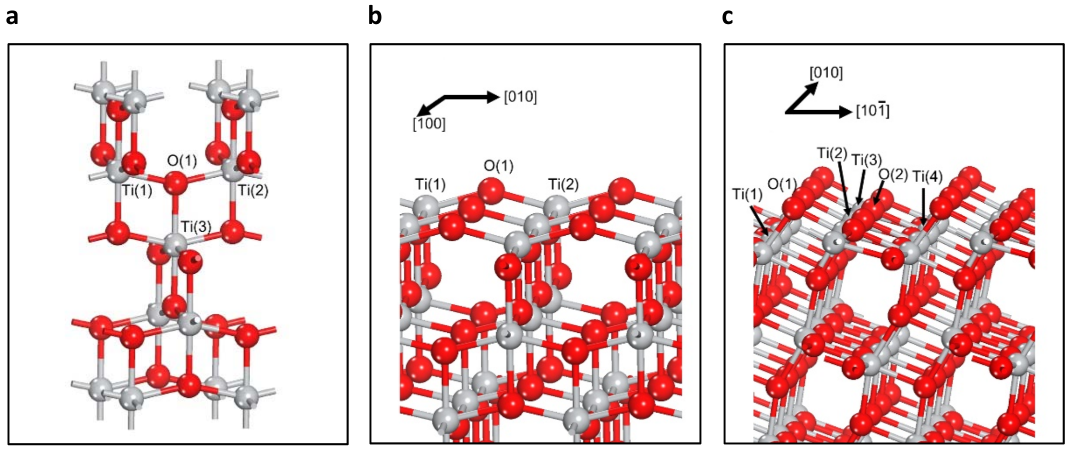

Supplementary Figure 7. Calculated structures of anatase TiO_2. a, bulk; **b**, unreconstructed clean TiO_2(001) surface; **c**, clean TiO_2(101) surface. The atoms are labeled for further discussion (Supplementary Table 3).

Supplementary Table 3. Bond distances and bond angles of calculated anatase TiO_2 structures. Corresponding structures are presented in Supplementary Fig. 7, which contain bulk anatase, unreconstructed clean anatase TiO_2(001) and anatase TiO_2(101) surface in sequence.

<table>
<tr><td colspan="8">Supplementary Fig. 7a</td></tr>
<tr><td>Ti(1)-O(1)</td><td>O(1)-Ti(2)</td><td>Ti(1)-O(1)</td><td>O(1)-Ti(3)</td><td>Ti(3)-O(1)</td><td>O(1)-Ti(2)</td></tr>
<tr><td>1.94 Å</td><td>1.94 Å</td><td>1.94 Å</td><td>1.97 Å</td><td>1.97 Å</td><td>1.94 Å</td></tr>
<tr><td colspan="2">∠Ti(1)-O(1)-Ti(2)</td><td colspan="2">∠Ti(1)-O(1)-Ti(3)</td><td colspan="2">∠Ti(3)-O(1)-Ti(2)</td></tr>
<tr><td colspan="2">156.0°</td><td colspan="2">102.0°</td><td colspan="2">102.0°</td></tr>
<tr><td colspan="6">Supplementary Fig. 7b</td></tr>
<tr><td colspan="3">Ti(1)-O(1)</td><td colspan="3">O(1)-Ti(2)</td></tr>
<tr><td colspan="3">1.96 Å</td><td colspan="3">1.96 Å</td></tr>
<tr><td colspan="6">∠Ti(1)-O(1)-Ti(2)</td></tr>
<tr><td colspan="6">149.8°</td></tr>
</table>

<table>
<tr><td colspan="8">Supplementary Fig. 7c</td></tr>
<tr><td>Ti(1)-O(1)</td><td>O(1)-Ti(2)</td><td>Ti(2)-O(2)</td><td>O(2)-Ti(3)</td><td>Ti(2)-O(2)</td><td>O(2)-Ti(4)</td><td>Ti(3)-O(2)</td><td>O(2)-Ti(4)</td></tr>
<tr><td>1.87 Å</td><td>1.85 Å</td><td>1.98 Å</td><td>1.98 Å</td><td>1.98 Å</td><td>2.01 Å</td><td>1.98 Å</td><td>2.01 Å</td></tr>
<tr><td colspan="2">∠Ti(1)-O(1)-Ti(2)</td><td colspan="2">∠Ti(2)-O(2)-Ti(3)</td><td colspan="2">∠Ti(2)-O(2)-Ti(4)</td><td colspan="2">∠Ti(3)-O(2)-Ti(4)</td></tr>
<tr><td colspan="2">99.8°</td><td colspan="2">146.7°</td><td colspan="2">100.6°</td><td colspan="2">100.7°</td></tr>
</table>

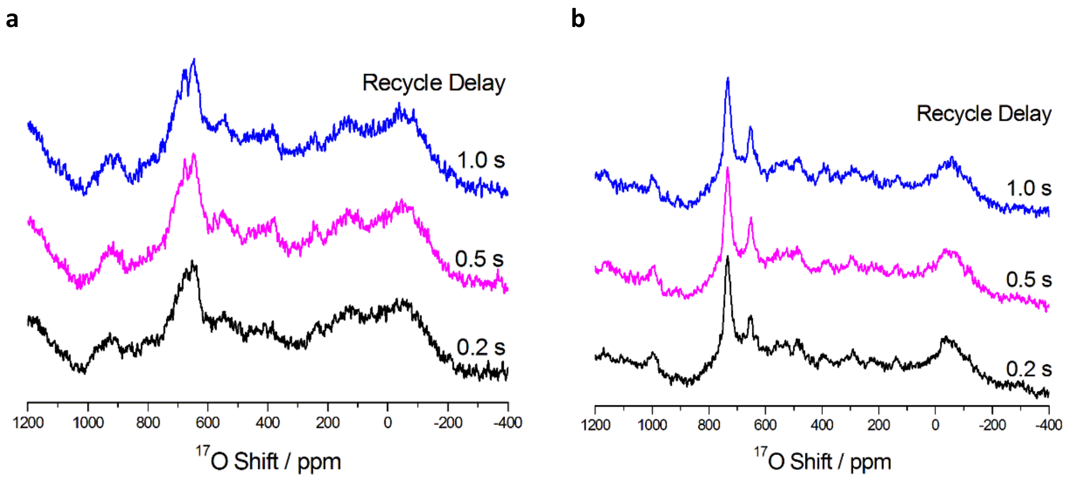

Supplementary Figure 8. Optimization of the recycle delays for faceted anatase TiO_2 samples. ^{17}O MAS NMR spectra of **a**, NS001-TiO_2 (2 h-vacuum dried) and **b**, NO101-TiO_2 (12 h-vacuum dried), at different recycle delays were acquired. A single pulse sequence with TPPM ^1H decoupling were used and the number of scans were set to be 800. It is clear that a recycle delay of 0.5 s is long enough to quantitatively measure the signals and thus 0.5 s was used as the recycle delay for obtaining the spectra shown in Fig. 1. The recycle delays for NF1-TiO_2 and NF2-TiO_2 samples were also optimized by using the same method.

Supplementary Table 4. Parameters for acquiring ^{17}O NMR spectra on the 9.4 T NMR spectrometer. The mass of each sample, the recycle delays, the number of scans and corresponding acquisition time are listed below.

Sample	Vacuum-drying time / h	Mass / mg	Recycle delay / s	Number of acquisitions	Acquisition time / h
**NS001-TiO_2**	0	112.7	0.2	120000	6.7
	2	100.8	0.5	120000	16.7
	12	93.4	0.5	120000	16.7
**NO101-TiO_2**	0	114.6	0.2	40000	2.2
	2	108.9	0.5	118000	16.4
	12	108.4	0.5	110000	15.3
**NF1-TiO_2**	/	110.9	50.0	1200	16.7
**NF2-TiO_2**	12	105.6	1.0	60000	16.7

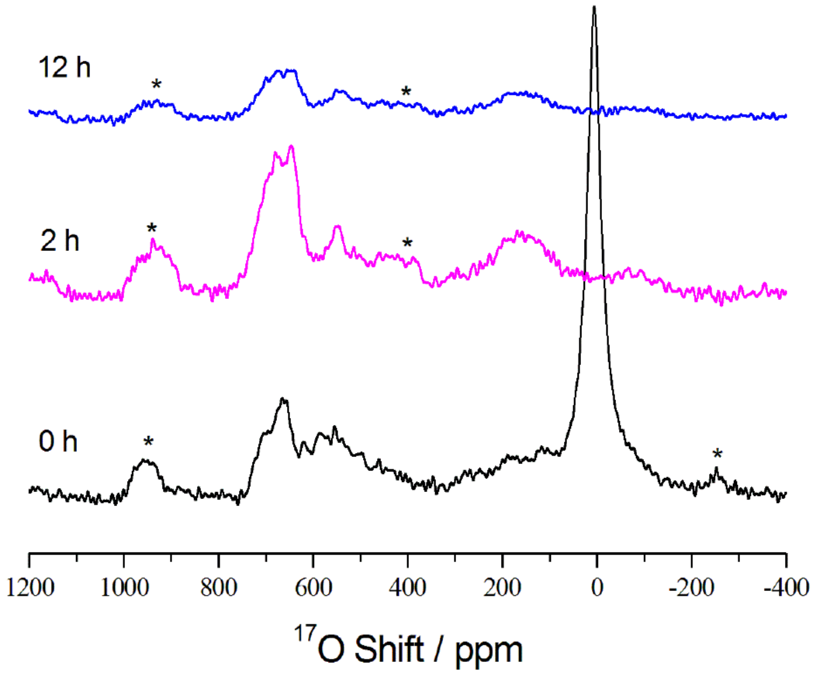

Supplementary Figure 9. ^{17}O NMR spectra of ^{17}O-labeled NS001-TiO_2 as a function of the vacuum-drying time. The spectra were obtained at 9.4 T under a MAS frequency of 14 kHz. The data was normalized according to the sample mass and the number of scans (Supplementary Table 4). Bottom to top: the sample that has adsorbed $H_2{}^{17}O$, with an estimated amount of 2.22 mg per 100 mg sample (Supplementary Fig. 20 and Supplementary Table 9); the ^{17}O-labeled sample which has been vacuum dried at room temperature for 2 h; the previous sample which has been vacuum dried at room temperature for another 10 h (the total drying time is 12 h). A rotor synchronized Hahn-echo sequence ($\pi/6$ - τ - $\pi/3$ - τ - acquisition) and an optimized recycle delay (Supplementary Table 4), with ^{1}H decoupling, were used. Asterisks denote sidebands.

Supplementary Note 2

There is a strong and relatively sharp peak at around 0 ppm for the hydrated NS001-TiO_2 sample without vacuum-drying. The amount of the adsorbed water on this sample (bottom spectrum in Supplementary Fig. 9) is calculated to be 1.9 molecules per 3.79 $\times$ 3.79 Å^2 (1.9 ML), using its ^{1}H NMR spectra, see Supplementary Fig. 20 and Supplementary Table 9). This exceeds the amount needed for a fully hydrated surface state (0.5 ML) (Supplementary Fig. 20). Therefore, the water in excess should be molecularly adsorbed. Since this resonance disappears after the sample was vacuum-dried for 2 hours, it is ascribed to molecularly adsorbed water. The resonant frequencies of other ^{17}O NMR signals of NS001-TiO_2 are independent of the time for vacuum-drying. In addition, hydrogen bond should have formed between the molecularly adsorbed water and the surface hydroxyl groups generated by dissociation of the initially adsorbed water. This can partially explain the reason why the peak of hydroxyl groups of the hydrated sample, centered at 150 ppm, has a smaller intensity than the

sample which was vacuum-dried for 2 hours.

The intensities of all ^{17}O signals decrease during the second room-temperature (RT) vacuum-drying process (from vacuum dried for 2 hours to 12 hours). This may arise from a variety processes, including the dehydroxylation of surface OH group, possible dynamic exchange of different surface sites (i.e., non-hydroxyl surface oxygen ions with hydroxyl groups), as well as isotopic exchange between surface oxygen ions and a small amount of unlabeled water that may enter the vacuum tube with prolonged process time.[4]

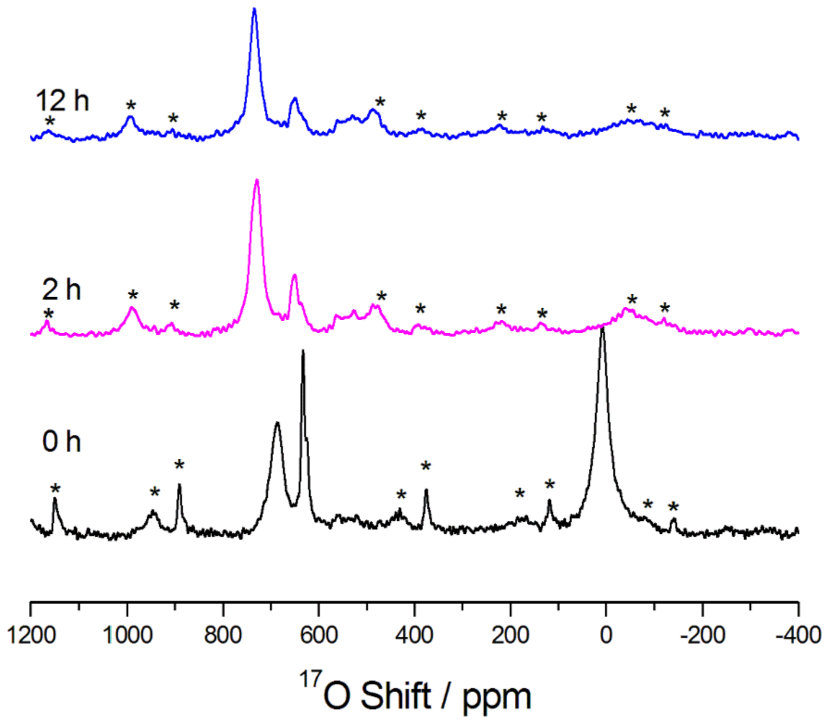

Supplementary Figure 10. Normalized ^{17}O NMR spectra of ^{17}O-labeled NO101-TiO_2 as a function of the vacuum-drying time. The spectra were obtained at 9.4 T under a MAS frequency of 14 kHz. Bottom to top: the sample that has adsorbed $H_2{}^{17}O$, with an estimated amount of 2.17 mg per 100 mg sample (Supplementary Fig. 20 and Supplementary Table 9); the sample which has been vacuum dried at room temperature for 2 h; the previous sample which has been vacuum dried at room temperature for another 10 h (the total drying time is 12 h). A rotor synchronized Hahn-echo sequence ($\pi/6$ - τ - $\pi/3$ - τ - acquisition) and optimized recycle delays (Supplementary Table 4), with ^{1}H decoupling, were used. The mass of the measured samples and the number of scans for acquiring each ^{17}O NMR spectrum are listed in Supplementary Table 4. Asterisks denote sidebands.

Supplementary Note 3

In the spectrum of the fully hydrated NO101-TiO_2 sample (bottom spectrum in Supplementary Fig. 10), the relatively sharp peak at around 0 ppm is assigned to molecularly adsorbed water according to its shift. Similar to that of NS001-TiO_2, the intensity of this peak decreases significantly when the sample was exposed to vacuum. Different from NS001-TiO_2, ^{17}O MAS NMR spectra of NO101-TiO_2 are more sensitive to the amount of the adsorbed water. The resonant frequencies and intensities of the signals vary when removing the adsorbed water by vacuum-drying at room temperature. After most of the adsorbed water is removed, the frequencies of these resonances do not change much with extended drying. The signal of this sample do not decrease much during the prolonged vacuum drying process, which may be associated with the lower activity of the {101} facets[5]. The O_{2c} signals of the fully hydrated sample, at 686 and 631 ppm, have lower chemical shifts than those of the vacuum-dried one. This may be

attributed to the influence of the hydrogen bond formed between the surface O_{2c} atoms and the molecularly adsorbed water, which has an electron-donating effect to the O_{2c} atoms.

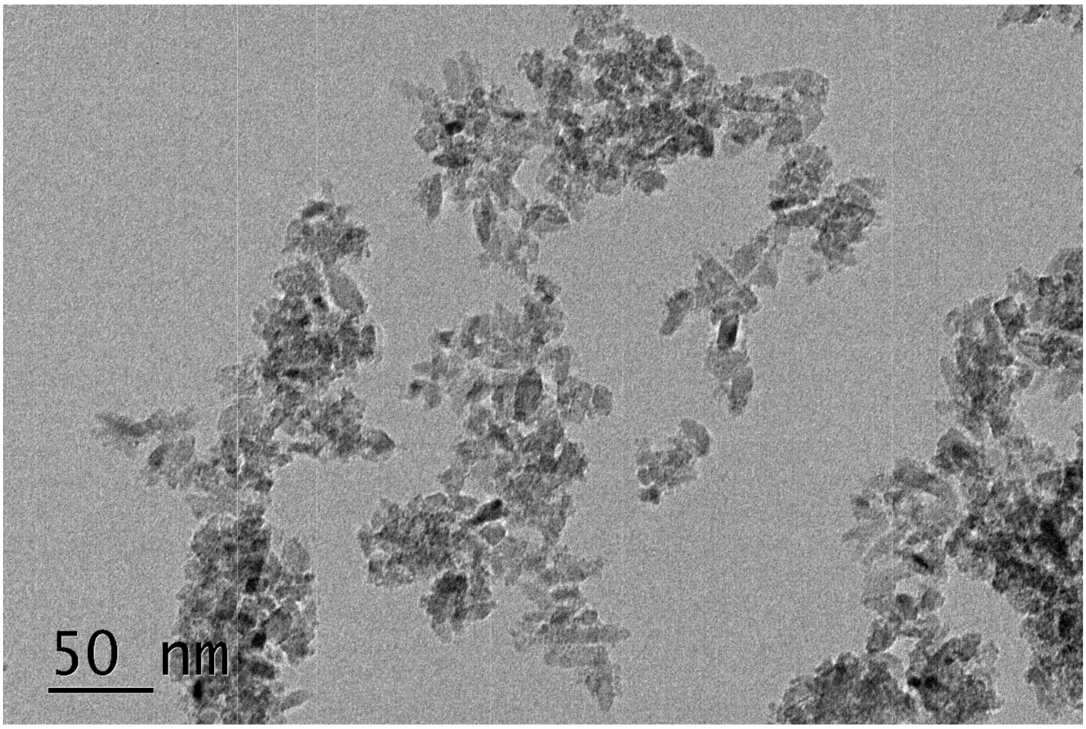

Supplementary Figure 11. TEM image of the non-faceted NF2-TiO_2 sample. The average size of the particles is about 10 nm.

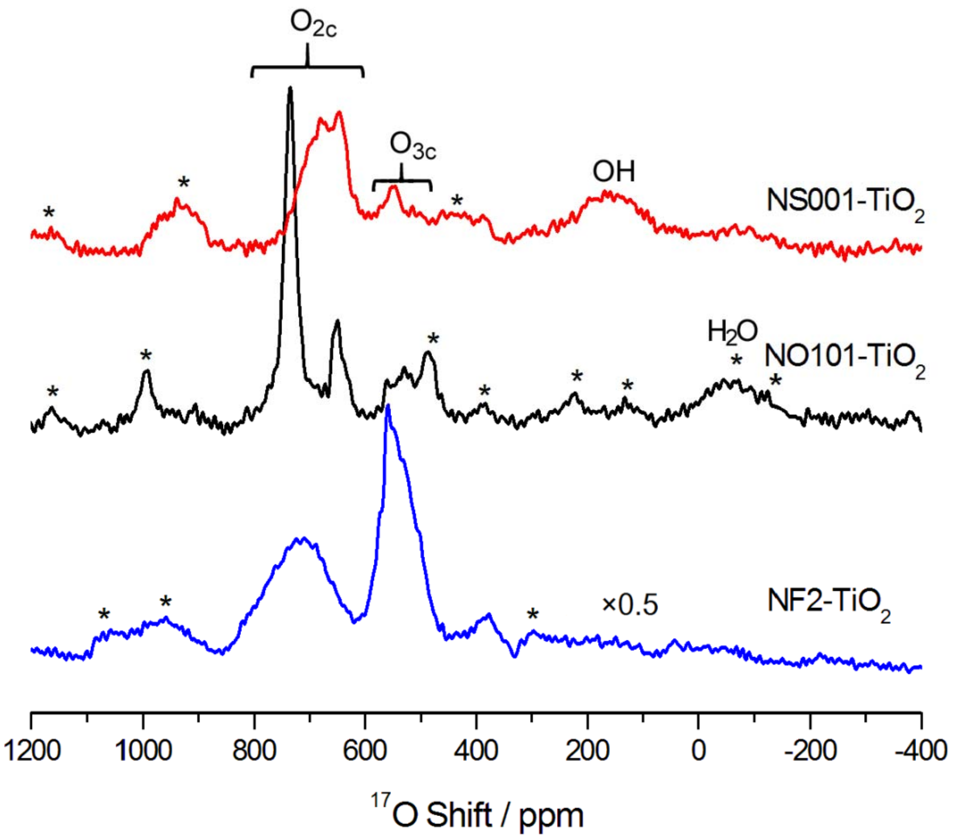

Supplementary Figure 12. ^{17}O NMR spectra of faceted anatase titania nanocrystals compared to a non-faceted sample with a large surface area. The intensities in the ^{17}O NMR spectra of NS001-TiO_2 (top, 2 h-vacuum dried), NO101-TiO_2 (middle, 12 h-vacuum dried) and non-faceted NF2-TiO_2 (bottom, 12 h-vacuum dried) are normalized according to the sample mass and the number of scans.

Supplementary Note 4

NF2-TiO_2, which has a surface area of 259 $m^2 \cdot g^{-1}$ (Supplementary Table 2), is confirmed as anatase TiO_2 with XRD (Supplementary Fig. 1). Since it was prepared with a very short hydrothermal time, 1.5 h, there was not enough time to form ordered surface structure.[1] Therefore, NF2-TiO_2 should be non-faceted nanoparticles, which is demonstrated by the TEM image (Supplementary Fig. 11), exhibiting irregular morphologies and coarse surfaces.

The ^{17}O NMR spectrum of non-faceted NF2-TiO_2 shows a much broader O_{2c} signal, which covers the frequency range of O_{2c} signals of NS001-TiO_2 and NO101-TiO_2, indicating more complicated surface environments originated from the presence of different facets, presumably including (001) and (101). The larger intensity of the O_{2c} and O_{3c} signals of NF2-TiO_2 can be ascribed to its much larger surface area (Supplementary Table 2). The small peak centered at 380 ppm of NF2-TiO_2 should arise from hydroxyl groups.

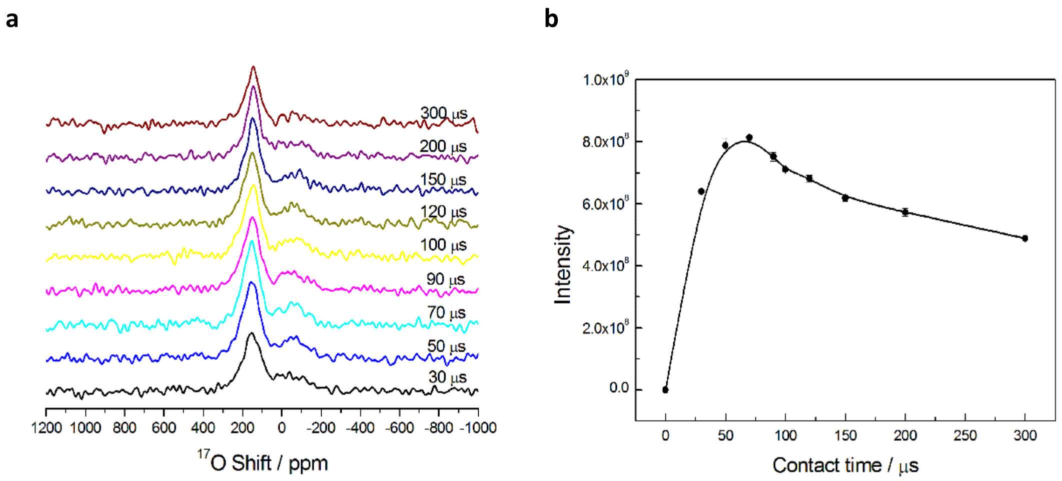

Supplementary Figure 13. $^{1}H \rightarrow ^{17}O$ CP-MAS NMR data of NS001-TiO_2. a, $^{1}H \rightarrow ^{17}O$ CP-MAS NMR spectra of 2h-vaccum-dried ^{17}O-labeled NS001-TiO_2, as a function of the contact time. **b**, The intensity of the peak centered at about 150 ppm in **a** as a function of contact time. The spectra were obtained at 9.4 T under a MAS frequency of 14 kHz. The optimized recycle delay of 1 s was used.

Supplementary Note 5

The CP intensity increases rapidly at short contact times and reaches a maximum at 70 μs, while it decreases with longer contact times. This CP behavior suggests a very large $^{1}H \rightarrow ^{17}O$ dipolar coupling present in oxygen ions directly bound to proton and has also been observed in many other materials[4, 6-8]. Therefore, this signal is assigned to rigid surface hydroxyl groups on NS001-TiO_2. In addition, a small peak centered at about -75 ppm is also observed, which can be attributed to the molecularly adsorbed water. The weaker intensity of this peak compared to the resonance due to hydroxyl groups in the CP spectra can be ascribed to the motion of the water species, which inevitably decreases the $^{1}H \rightarrow ^{17}O$ dipolar coupling in the molecularly adsorbed water.

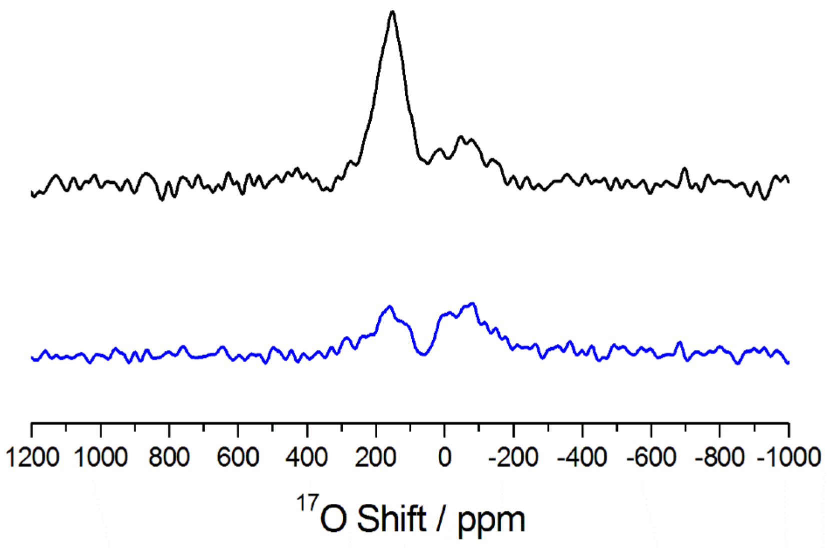

Supplementary Figure 14. $^{1}H \rightarrow {}^{17}O$ CP-MAS NMR spectra of the two faceted samples. The contact time is 70 μs. Top: 2 h-vaccum-dried NS001-TiO_2; bottom: 2 h-vaccum-dried NO101-TiO_2.

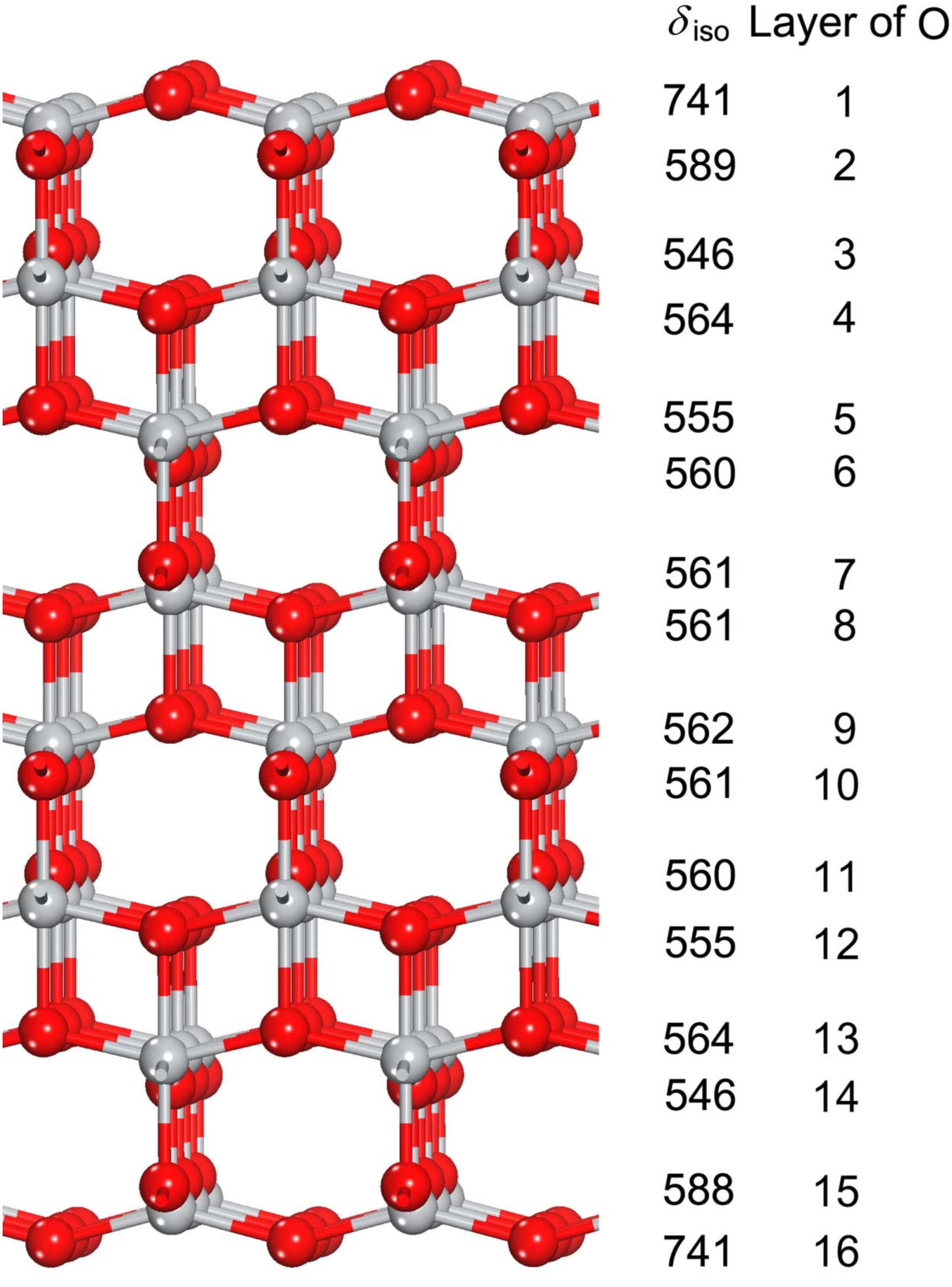

Supplementary Figure 15. Calculated structure of unreconstructed clean anatase TiO_2(001). This structure is denoted as CL in the text. Isotropic chemical shifts δ_{iso} of the oxygen species in each layer are listed, for which δ_{ref} = 60.

Supplementary Table 5. Calculated NMR parameters for oxygen species in the unreconstructed clean anatase TiO_2(001). Isotropic chemical shifts (δ_{iso}), quadrupolar parameters (C_Q and η) and center of gravity (δ_{CG}) of the NMR signals are listed below. δ_{ref} = 60. δ_{CG} is calculated according to Lippmaa[9]. Corresponding structure has been presented in Supplementary Fig. 15.

	δ_{iso}/ppm	C_Q/MHz	η	δ_{CG}/ppm	Assignment
**1**	741	2.19	0.46	731	Surface O_{2c}
**2**	589	1.40	0.48	585	Surface O_{3c}
**3**	546	1.78	0.04	540	O_{3c}
**4**	564	1.28	0.18	561	O_{3c}
**5**	555	1.21	0.24	552	O_{3c}
**6**	560	1.23	0.38	557	O_{3c}
**7**	561	1.26	0.35	558	O_{3c}
**8**	561	1.26	0.39	558	O_{3c}
**9**	562	1.25	0.40	559	O_{3c}
**10**	561	1.26	0.36	558	O_{3c}
**11**	560	1.23	0.38	557	O_{3c}
**12**	555	1.21	0.25	552	O_{3c}
**13**	564	1.28	0.18	561	O_{3c}
**14**	546	1.78	0.04	540	O_{3c}
**15**	588	1.40	0.49	584	Surface O_{3c}
**16**	741	2.18	0.46	731	Surface O_{2c}

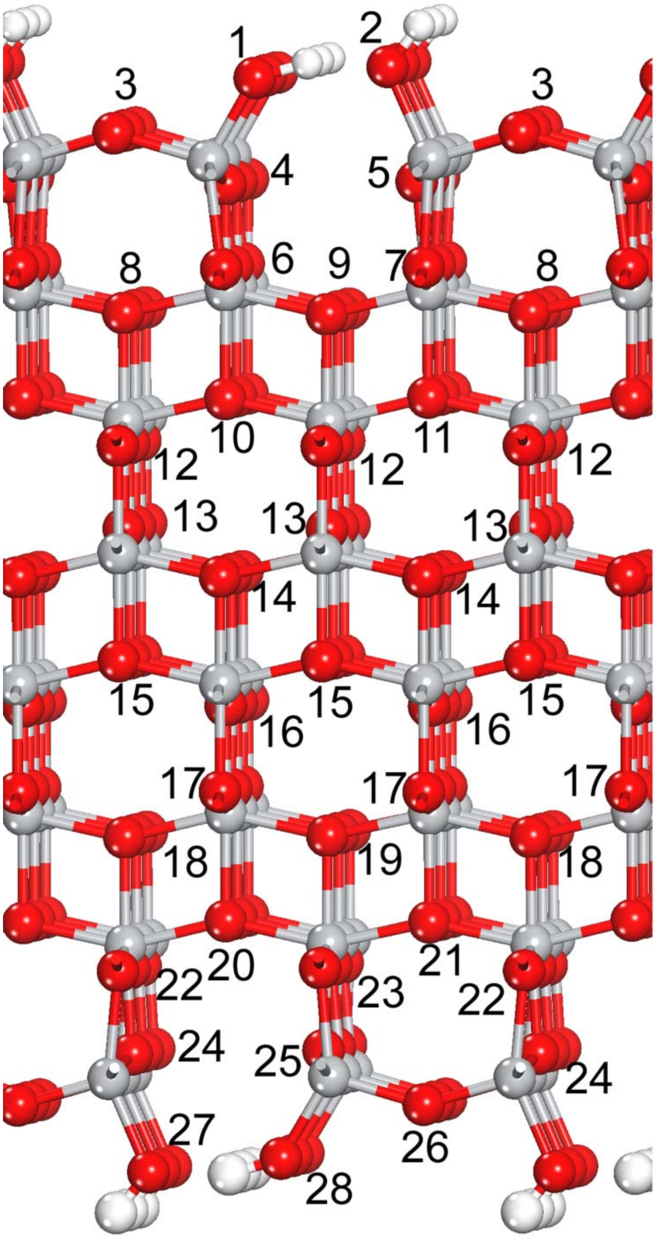

δ_{iso}	No. of O
430 235	1 2
627	3
533 529	4 5
542 542	6 7
551 559	8 9
558 566	10 11
561	12
561	13
561	14
561	15
560	16
561	17
565 557	18 19
558 549	20 21
542 540	22 23
528 532	24 25
625	26
236 423	27 28

Supplementary Figure 16. Calculated structure of hydrated anatase TiO_2(001). In this model, water dissolves on the surface at a coverage of $^1/_2$ ML. This structure is denoted as DA in the text. Isotropic chemical shifts δ_{iso} of the oxygen species in each layer are listed, for which δ_{ref} = 59.

Supplementary Table 6. Calculated NMR parameters for oxygen species in hydrated anatase TiO_2(001). Isotropic chemical shifts (δ_{iso}), quadrupolar parameters (C_Q and η) and center of gravity (δ_{CG}) of the NMR signals are listed below. δ_{ref} = 59. Water dissolves on this surface at a coverage of $^1/_2$ ML, as described in Supplementary Fig. 16.

	δ_{iso}/ppm	C_Q/MHz	η	δ_{CG}/ppm	Assignment
**1**	430	3.57	0.33	403	OH
**2**	235	7.06	0.26	131	OH
**3**	627	1.28	0.18	624	Surface O_{2c}
**4**	533	1.08	0.88	530	Surface O_{3c}
**5**	529	1.29	0.71	525	Surface O_{3c}
**6**	542	1.24	0.85	538	O_{3c}
**7**	542	1.54	0.60	537	O_{3c}
**8**	551	1.21	0.65	548	O_{3c}
**9**	559	1.24	0.30	556	O_{3c}
**10**	558	1.34	0.24	554	O_{3c}
**11**	566	1.20	0.40	563	O_{3c}
**12**	561	1.25	0.40	558	O_{3c}
**13**	561	1.25	0.40	558	O_{3c}
**14**	561	1.25	0.38	558	O_{3c}
**15**	561	1.25	0.38	558	O_{3c}
**16**	560	1.25	0.40	557	O_{3c}
**17**	561	1.24	0.41	558	O_{3c}
**18**	565	1.18	0.40	562	O_{3c}
**19**	557	1.35	0.25	553	O_{3c}
**20**	558	1.23	0.34	555	O_{3c}
**21**	549	1.19	0.67	546	O_{3c}
**22**	542	1.51	0.64	537	O_{3c}
**23**	540	1.22	0.87	536	O_{3c}
**24**	528	1.25	0.80	524	Surface O_{3c}
**25**	532	1.10	0.88	529	Surface O_{3c}
**26**	625	1.29	0.20	622	Surface O_{2c}
**27**	236	7.05	0.26	132	OH
**28**	423	3.60	0.34	396	OH

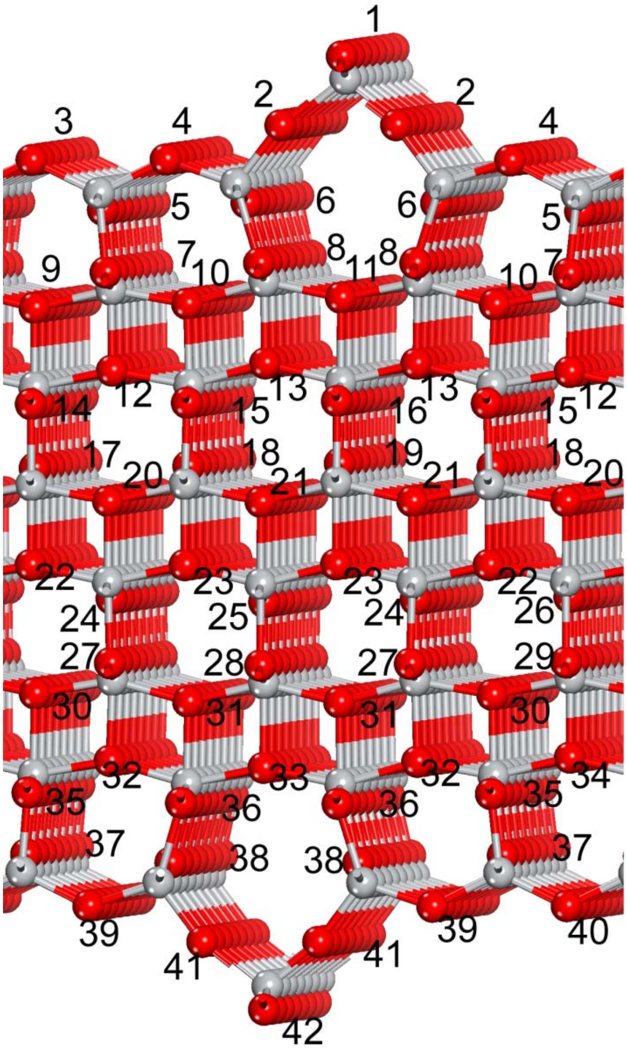

δ_{iso}			No. of O		
714			1		
656			2		
694	674		3	4	
543	545		5	6	
528	555		7	8	
539	552	578	9	10	11
556	567		12	13	
554	560	566	14	15	16
560	562	558	17	18	19
558	567		20	21	
558	567		22	23	
562	558	560	24	25	26
560	566	554	27	28	29
556	567		30	31	
552	577	539	32	33	34
554	528		35	36	
545	543		37	38	
673	693		39	40	
656			41		
714			42		

Supplementary Figure 17. Calculated structure of 1×4-reconstructed clean anatase TiO_2(001). This structure is denoted as RC-CL in the text. Isotropic chemical shifts δ_{iso} of the oxygen species in each layer are listed, for which δ_{ref} = 60.

Supplementary Table 7. Calculated NMR parameters for oxygen species in 1×4-reconstructed clean anatase TiO_2(001). Isotropic chemical shifts (δ_{iso}), quadrupolar parameters (C_Q and η) and center of gravity (δ_{CG}) of the NMR signals are listed below. δ_{ref} = 60. Corresponding structure is described in Supplementary Fig. 17.

	δ_{iso}/ppm	C_Q/MHz	η	δ_{CG}/ppm	Assignment
**1**	714	1.85	0.08	707	Surface O_{2c}
**2**	656	1.66	0.53	650	Surface O_{2c}
**3**	694	1.34	0.47	690	Surface O_{2c}
**4**	674	1.38	0.28	670	Surface O_{2c}
**5**	543	1.04	0.60	541	Surface O_{3c}
**6**	545	1.07	0.61	542	Surface O_{3c}
**7**	528	1.34	0.41	524	O_{3c}
**8**	555	1.25	0.93	551	O_{3c}
**9**	539	1.14	0.04	536	O_{3c}
**10**	552	1.32	0.01	548	O_{3c}
**11**	578	1.23	0.89	574	O_{3c}
**12**	556	1.36	0.15	552	O_{3c}
**13**	567	1.16	0.47	564	O_{3c}
**14**	554	1.17	0.24	551	O_{3c}
**15**	560	1.24	0.35	557	O_{3c}
**16**	566	1.32	0.57	562	O_{3c}
**17**	560	1.23	0.24	557	O_{3c}
**18**	562	1.25	0.38	559	O_{3c}
**19**	558	1.26	0.60	554	O_{3c}
**20**	558	1.27	0.48	554	O_{3c}
**21**	567	1.27	0.29	564	O_{3c}
**22**	558	1.26	0.48	555	O_{3c}
**23**	567	1.28	0.29	564	O_{3c}
**24**	562	1.25	0.38	559	O_{3c}
**25**	558	1.26	0.61	554	O_{3c}
**26**	560	1.22	0.24	557	O_{3c}
**27**	560	1.24	0.35	557	O_{3c}
**28**	566	1.31	0.58	562	O_{3c}
**29**	554	1.17	0.23	551	O_{3c}
**30**	556	1.36	0.14	552	O_{3c}
**31**	567	1.16	0.46	564	O_{3c}
**32**	552	1.32	0.02	548	O_{3c}
**33**	577	1.24	0.86	573	O_{3c}
**34**	539	1.13	0.02	536	O_{3c}
**35**	554	1.25	0.93	550	O_{3c}
**36**	528	1.36	0.40	524	O_{3c}
**37**	545	1.07	0.61	542	Surface O_{3c}
**38**	543	1.04	0.62	541	Surface O_{3c}
**39**	673	1.39	0.27	669	Surface O_{2c}
**40**	693	1.36	0.46	689	Surface O_{2c}
**41**	656	1.64	0.54	650	Surface O_{2c}
**42**	714	1.85	0.08	707	Surface O_{2c}

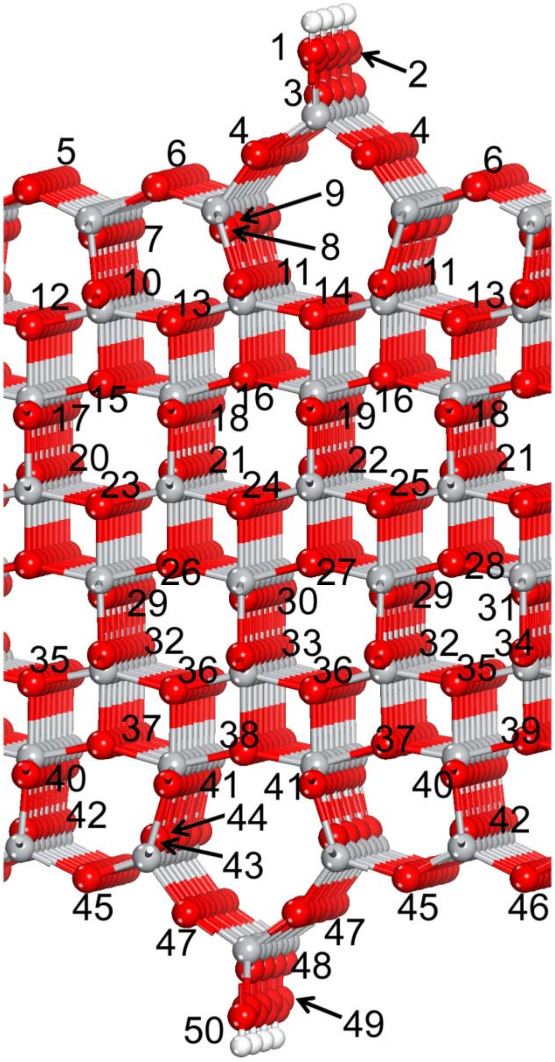

δ_{iso}			No. of O		
207	408		1	2	
609			3		
614			4		
692	669		5	6	
541	552	536	7	8	9
526	553		10	11	
539	552	579	12	13	14
556	567		15	16	
555	561	567	17	18	19
560	562	558	20	21	22
558	571	565	23	24	25
558	565	571	26	27	28
562	559	561	29	30	31
563	567	555	32	33	34
556	567		35	36	
552	579	538	37	38	39
553	526		40	41	
553	536	541	42	43	44
668	692		45	46	
614			47		
609			48		
408	207		49	50	

Supplementary Figure 18. Calculated structure of hydrated 1×4-reconstructed anatase TiO_2(001). In this model, water dissolves on the surface and this structure is denoted as RC-DA in the text. Isotropic chemical shifts δ_{iso} of the oxygen species in each layer are listed, for which δ_{ref} = 59.

Supplementary Table 8. Calculated NMR parameters for oxygen species in hydrated 1×4-reconstructed anatase TiO_2(001). Isotropic chemical shifts (δ_{iso}), quadrupolar parameters (C_Q and η) and center of gravity (δ_{CG}) of the NMR signals are listed below. δ_{ref} =59. Corresponding structure is described in Supplementary Fig. 18.

	δ_{iso}/ppm	C_Q/MHz	η	δ_{CG}/ppm	Assignment
**1**	207	6.94	0.39	104	OH
**2**	408	3.95	0.27	375	OH
**3**	609	1.02	0.42	607	Surface O_{2c}
**4**	614	1.41	0.38	610	Surface O_{2c}
**5**	692	1.31	0.45	688	Surface O_{2c}
**6**	669	1.37	0.21	665	Surface O_{2c}
**7**	541	1.04	0.58	539	Surface O_{3c}
**8**	552	1.08	0.54	549	Surface O_{3c}
**9**	536	1.00	0.91	533	Surface O_{3c}
**10**	526	1.33	0.43	522	O_{3c}
**11**	553	1.31	0.86	549	O_{3c}
**12**	539	1.14	0.03	536	O_{3c}
**13**	552	1.33	0.09	548	O_{3c}
**14**	579	1.24	0.90	575	O_{3c}
**15**	556	1.38	0.14	552	O_{3c}
**16**	567	1.16	0.47	564	O_{3c}
**17**	555	1.17	0.23	552	O_{3c}
**18**	561	1.25	0.34	558	O_{3c}
**19**	567	1.33	0.57	563	O_{3c}
**20**	560	1.24	0.22	557	O_{3c}
**21**	562	1.26	0.37	559	O_{3c}
**22**	558	1.26	0.62	554	O_{3c}
**23**	558	1.27	0.47	554	O_{3c}
**24**	571	1.24	0.30	568	O_{3c}
**25**	565	1.32	0.27	561	O_{3c}
**26**	558	1.33	0.44	554	O_{3c}
**27**	565	1.33	0.29	561	O_{3c}
**28**	571	1.24	0.28	568	O_{3c}
**29**	563	1.26	0.36	560	O_{3c}
**30**	559	1.27	0.61	555	O_{3c}
**31**	561	1.23	0.22	558	O_{3c}
**32**	561	1.25	0.35	558	O_{3c}
**33**	567	1.32	0.58	563	O_{3c}
**34**	555	1.18	0.21	552	O_{3c}
**35**	556	1.39	0.14	552	O_{3c}
**36**	567	1.16	0.47	564	O_{3c}
**37**	552	1.34	0.09	548	O_{3c}
**38**	579	1.25	0.88	575	O_{3c}
**39**	538	1.14	0.02	535	O_{3c}

**40**	553	1.30	0.86	549	O_{3c}
**41**	526	1.34	0.42	522	O_{3c}
**42**	553	1.08	0.55	550	Surface O_{3c}
**43**	536	1.00	0.91	533	Surface O_{3c}
**44**	541	1.03	0.59	539	Surface O_{3c}
**45**	668	1.38	0.21	664	Surface O_{2c}
**46**	692	1.32	0.45	688	Surface O_{2c}
**47**	614	1.40	0.38	610	Surface O_{2c}
**48**	609	1.02	0.42	607	Surface O_{2c}
**49**	408	3.97	0.28	375	OH
**50**	207	6.94	0.39	104	OH

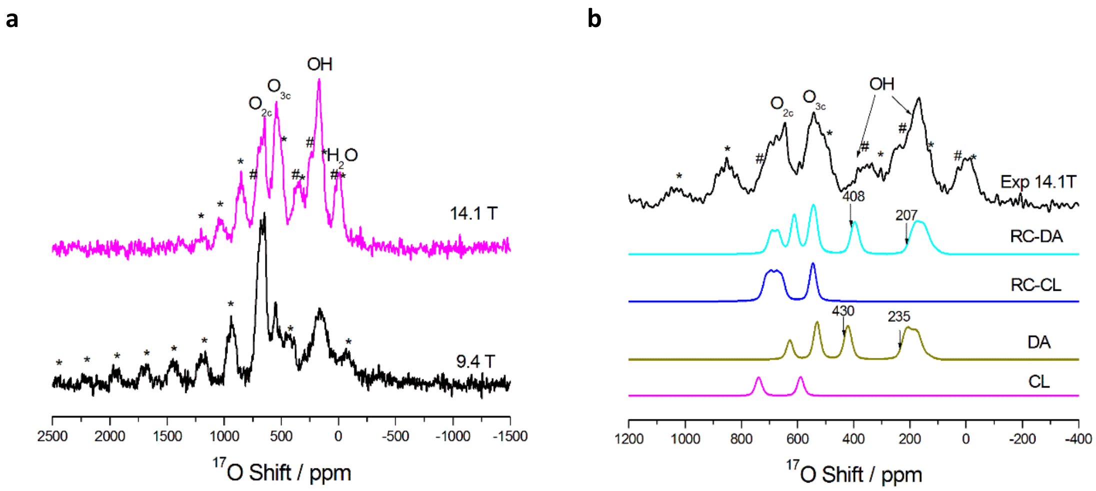

Supplementary Figure 19. ^{17}O NMR spectra of NS001-TiO_2 obtained at different external magnetic field strengths. a, Comparison of the ^{17}O spin-echo NMR spectra of 2 h-vacuum-dried NS001-TiO_2 obtained by NMR spectrometers of 9.4 T (bottom) and 14.1 T (top), respectively. A rotor synchronized Hahn-echo sequence ($\pi/6$ - τ - $\pi/3$ - τ - acquisition) and optimized enough recycle delay of 0.5 s were used. The sample was packed into a 4 mm zirconia rotor, and NMR spectra were obtained at a spinning rate of 14 kHz. 120000 (for 9.4 T) and 84000 (for 14.1 T) scans were acquired, respectively. **b**, Comparison of the experimental data measured at 14.1 T with the simulated spectra according to DFT calculations using the same structural models in Fig. 2. Isotropic chemical shifts (δ_{iso}) of oxygen in the hydroxyl groups have been marked. Asterisks denote sidebands of the O_{2c} signal, and #s denote sidebands of the O_{3c} signal.

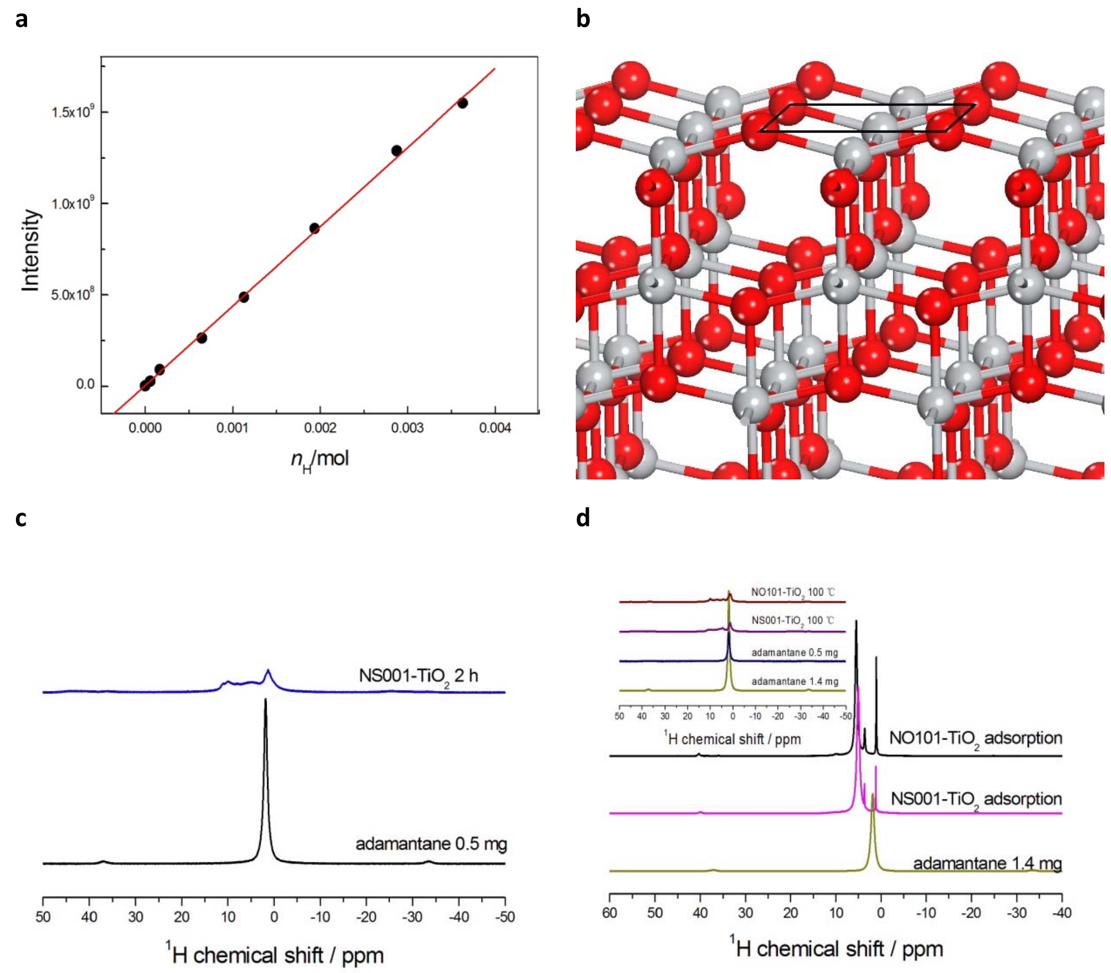

Supplementary Figure 20. Determining water content on the samples by interpolation method using adamantane as the reference material. a, The intensity of ^{1}H signal as a function of the amount of H in adamantane. The integral range is set to -40 ~ 40 ppm. The number of scans collected is 16. The amount of H is calculated according to the mass of the measured adamantane. A recycle delay of 10 s is used to ensure quantitative measurement. The linear correlation of the intensity (*y*) of the spectra and the content of ^{1}H atoms (*x*/mol) is $y=5.54\times10^6+4.34\times10^{11}x$. **b**, Calculated structure of unreconstructed clean anatase TiO_2(001), where the square encloses a unit area, 3.79×3.79 Å^2, which contains one Ti_{5c} atom. A fully hydrated surface state means each two Ti_{5c} atoms adsorb one water molecule, i.e. the coverage of water is 0.5 molecular layer (ML). **c**, Comparison of the ^{1}H NMR spectrum of the 2 h-vacuum-dried NS001-TiO_2 to that of adamantane (0.5 mg). The measured mass of 2 h-vacuum-dried NS001-TiO_2 is 100.8 mg. BET surface area of this sample is 67 $m^2\cdot g^{-1}$, and intensity of the 16-scan ^{1}H NMR spectrum is 2.60×10^7 a. u., so the number of water molecules adsorbed on unit area of the surface is estimated to be 0.30. **d**, Comparison of the ^{1}H NMR spectra of other four samples to those of the adamantane. The content of the adsorbed water is calculated and listed in Supplementary Table 9.

Supplementary Table 9. Water content of the samples. The method is present in Supplementary Fig. 20.

**Sample**		**Mass / mg**	**Intensity of 16 scans / 10^8 a.u.**	**Water on 100 mg sample / mg**	**$H_2{}^{17}O$ adsorbed on 100 mg sample / mg**	**ML of the adsorbed water***
**NS001-TiO_2**	Dried at 100 °C	91.0	0.26	0.46		
	Adsorbed $H_2{}^{17}O$	112.7	1.51	2.68	2.22	1.9
	2 h-RT-vacuum dried	108.4	0.26	0.42		0.3
**NO101-TiO_2**	Dried at 100 °C	108.7	0.32	0.50		
	Adsorbed $H_2{}^{17}O$	114.6	1.53	2.67	2.17	

*The number of adsorbed water molecules onto a unit area, 3.79×3.79 Å, which contains one Ti_{5c} atom on unreconstructed TiO_2(001) surface.

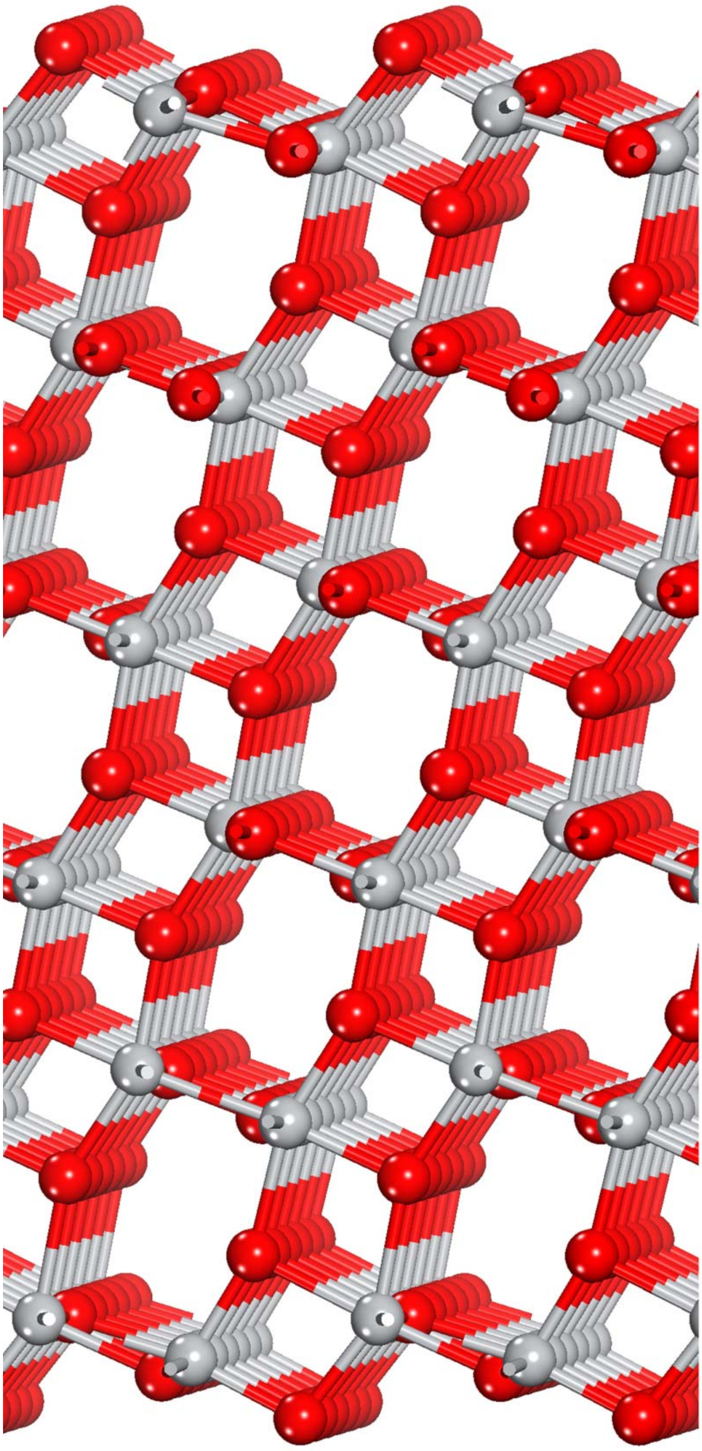

δ_{iso}	Layer of O
773	1
503	2
535	3
559	4
560	5
548	6
561	7
564	8
560	9
559	10
561	11
561	12
561	13
561	14
559	15
560	16
563	17
560	18
549	19
560	20
559	21
536	22
503	23
774	24

Supplementary Figure 21. Calculated structure of clean anatase TiO_2(101). This structure is denoted as CL in the text. Isotropic chemical shifts δ_{iso} of the oxygen species in each layer are listed, for which δ_{ref} = 54.

Supplementary Table 10. Calculated NMR parameters for oxygen species in clean anatase TiO_2(101). Isotropic chemical shifts (δ_{iso}), quadrupolar parameters (C_Q and η) and center of gravity (δ_{CG}) of the NMR signals are listed below. δ_{ref} =54. Corresponding structure is described in Supplementary Fig. 21.

	**δ_{iso}/ppm**	**C_Q/MHz**	**η**	**δ_{CG}/ppm**	**Assignment**
**1**	773	1.28	0.67	769	Surface O_{2c}
**2**	503	1.33	0.82	499	Surface O_{3c}
**3**	535	1.53	0.95	529	Surface O_{3c}
**4**	559	1.10	0.97	556	O_{3c}
**5**	560	1.77	0.27	553	O_{3c}
**6**	548	1.25	0.54	545	O_{3c}
**7**	561	1.25	0.60	557	O_{3c}
**8**	564	1.08	0.79	561	O_{3c}
**9**	560	1.47	0.35	555	O_{3c}
**10**	559	1.26	0.40	556	O_{3c}
**11**	561	1.26	0.40	558	O_{3c}
**12**	561	1.29	0.49	557	O_{3c}
**13**	561	1.29	0.49	557	O_{3c}
**14**	561	1.26	0.41	558	O_{3c}
**15**	559	1.26	0.39	556	O_{3c}
**16**	560	1.48	0.36	555	O_{3c}
**17**	563	1.08	0.78	560	O_{3c}
**18**	560	1.25	0.60	556	O_{3c}
**19**	549	1.26	0.54	545	O_{3c}
**20**	560	1.78	0.27	553	O_{3c}
**21**	559	1.10	0.97	556	O_{3c}
**22**	536	1.52	0.96	530	Surface O_{3c}
**23**	503	1.33	0.83	499	Surface O_{3c}
**24**	774	1.28	0.66	770	Surface O_{2c}

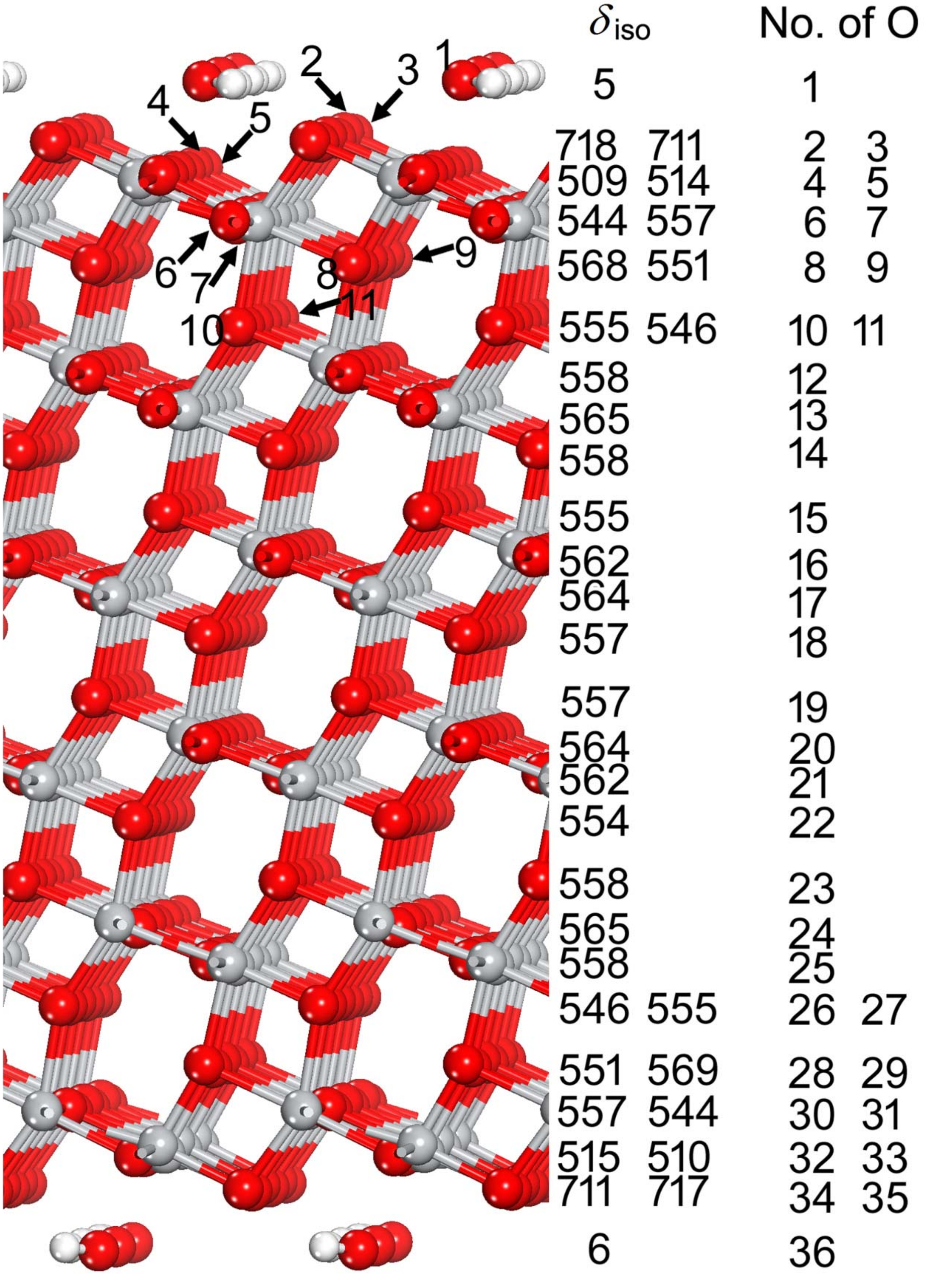

Supplementary Figure 22. Calculated structure of hydrated anatase TiO_2(101) with water molecularly adsorbed. In this model, the water coverage is $^1/_2$ ML, which means two surface Ti_{5c} sites adsorb one water molecule. This structure is denoted as MA in the text. Isotropic chemical shifts δ_{iso} of the oxygen species in each layer are listed, for which δ_{ref} = 56.

Supplementary Table 11. Calculated NMR parameters for oxygen species in hydrated anatase TiO_2(101) with water molecularly adsorbed. Isotropic chemical shifts (δ_{iso}), quadrupolar parameters (C_Q and η) and center of gravity (δ_{CG}) of the NMR signals are listed below. δ_{ref} =56. Corresponding structure is described in Supplementary Fig. 22.

	δ_{iso}/ppm	C_Q/MHz	η	δ_{CG}/ppm	Assignment
**1**	5	8.14	0.73	-154	Water
**2**	718	1.14	0.52	715	Surface O_{2c}
**3**	711	1.28	0.30	708	Surface O_{2c}
**4**	509	1.37	0.84	504	Surface O_{3c}
**5**	514	1.38	0.71	509	Surface O_{3c}
**6**	544	1.60	0.55	538	Surface O_{3c}
**7**	557	1.69	0.90	550	Surface O_{3c}
**8**	568	1.02	0.76	565	O_{3c}
**9**	551	1.22	0.87	547	O_{3c}
**10**	555	1.64	0.37	549	O_{3c}
**11**	546	1.79	0.31	539	O_{3c}
**12**	558	1.25	0.39	555	O_{3c}
**13**	565	1.25	0.43	562	O_{3c}
**14**	558	1.18	0.67	555	O_{3c}
**15**	555	1.43	0.41	551	O_{3c}
**16**	562	1.25	0.34	559	O_{3c}
**17**	564	1.25	0.34	561	O_{3c}
**18**	557	1.32	0.49	553	O_{3c}
**19**	557	1.31	0.50	553	O_{3c}
**20**	564	1.24	0.34	561	O_{3c}
**21**	562	1.25	0.33	559	O_{3c}
**22**	554	1.45	0.40	549	O_{3c}
**23**	558	1.17	0.69	555	O_{3c}
**24**	565	1.25	0.44	562	O_{3c}
**25**	558	1.24	0.40	555	O_{3c}
**26**	546	1.80	0.32	539	O_{3c}
**27**	555	1.66	0.38	549	O_{3c}
**28**	551	1.22	0.88	547	O_{3c}
**29**	569	1.07	0.71	566	O_{3c}
**30**	557	1.69	0.91	550	Surface O_{3c}
**31**	544	1.59	0.58	538	Surface O_{3c}
**32**	515	1.39	0.71	510	Surface O_{3c}
**33**	510	1.38	0.82	505	Surface O_{3c}
**34**	711	1.27	0.27	708	Surface O_{2c}
**35**	717	1.13	0.50	714	Surface O_{2c}
**36**	6	8.14	0.72	-152	Water

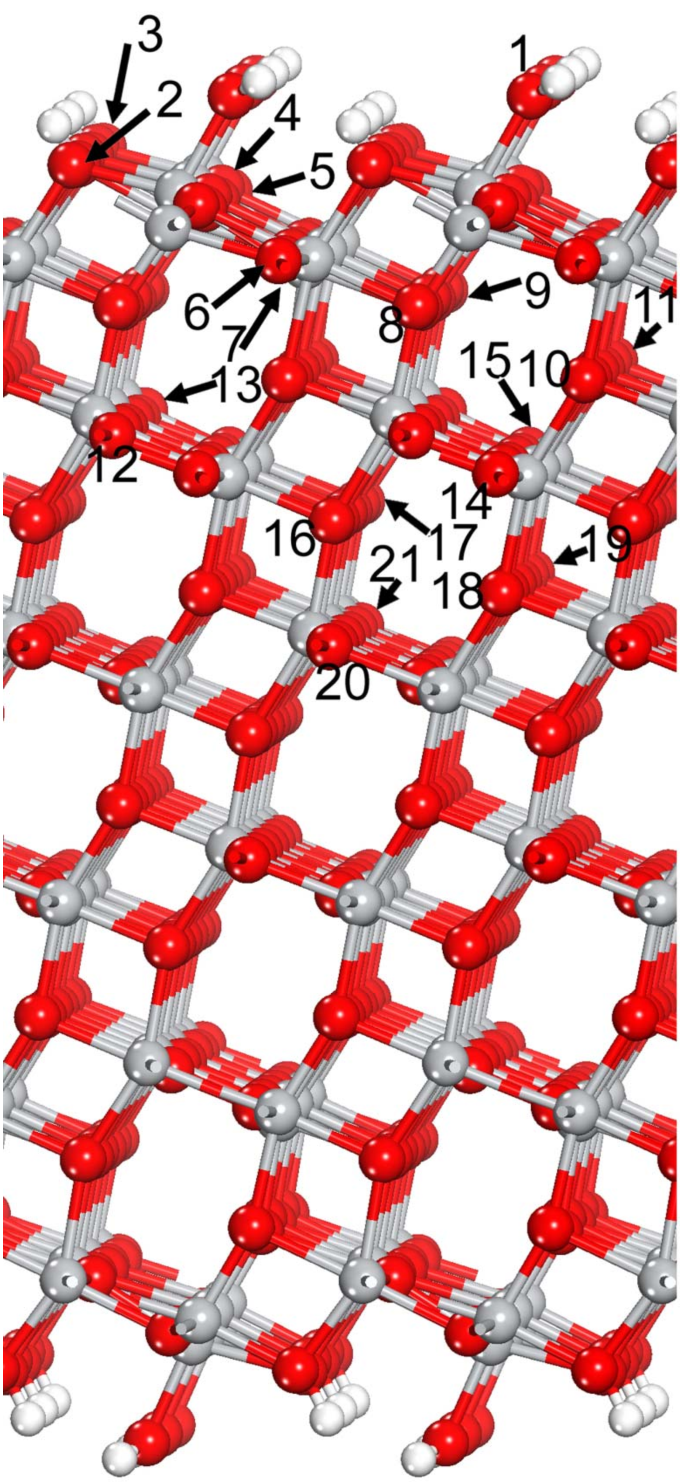

δ_{iso}		No. of O	
328		1	
734	200	2	3
527	552	4	5
557	549	6	7
605	543	8	9
574	538	10	11
560	564	12	13
569	559	14	15
561	556	16	17
561	553	18	19
563	568	20	21
565		22	
557		23	
557		24	
564		25	
568	563	26	27
553	561	28	29
557	561	30	31
559	568	32	33
565	560	34	35
538	575	36	37
544	606	38	39
550	557	40	41
552	527	42	43
202	734	44	45
333		46	

Supplementary Figure 23. Calculated structure of hydrated anatase TiO_2(101) with water dissociatively adsorbed. In this model, the water coverage is $^1/_2$ ML. This structure is denoted as DA in the text. Isotropic chemical shifts δ_{iso} of the oxygen species in each layer are listed, for which δ_{ref} = 57.

Supplementary Table 12. Calculated NMR parameters for oxygen species in hydrated anatase TiO_2(101) with water dissociatively adsorbed. Isotropic chemical shifts (δ_{iso}), quadrupolar parameters (C_Q and η) and center of gravity (δ_{CG}) of the NMR signals are listed below. δ_{ref} =57. Corresponding structure is described in Supplementary Fig. 23.

	δ_{iso}/ppm	C_Q/MHz	η	δ_{CG}/ppm	Assignment
**1**	328	6.34	0.11	246	OH
**2**	734	0.56	0.45	733	Surface O_{2c}
**3**	200	6.21	0.52	114	OH
**4**	527	1.50	0.71	522	Surface O_{3c}
**5**	552	1.13	0.70	549	Surface O_{3c}
**6**	557	1.42	0.50	553	Surface O_{3c}
**7**	549	1.69	0.84	542	Surface O_{3c}
**8**	605	0.70	0.60	604	O_{3c}
**9**	543	1.63	0.61	537	O_{3c}
**10**	574	1.20	0.57	571	O_{3c}
**11**	538	1.72	0.66	531	O_{3c}
**12**	560	1.30	0.45	556	O_{3c}
**13**	564	1.21	0.31	561	O_{3c}
**14**	569	1.22	0.34	566	O_{3c}
**15**	559	1.29	0.45	555	O_{3c}
**16**	561	1.22	0.43	558	O_{3c}
**17**	556	1.26	0.71	552	O_{3c}
**18**	561	1.25	0.46	558	O_{3c}
**19**	553	1.44	0.52	548	O_{3c}
**20**	563	1.24	0.32	560	O_{3c}
**21**	568	1.24	0.33	565	O_{3c}
**22**	565	1.24	0.32	562	O_{3c}
**23**	557	1.35	0.48	553	O_{3c}
**24**	557	1.35	0.42	553	O_{3c}
**25**	564	1.25	0.32	561	O_{3c}
**26**	568	1.24	0.32	565	O_{3c}
**27**	563	1.25	0.38	560	O_{3c}
**28**	553	1.44	0.55	548	O_{3c}
**29**	561	1.24	0.47	558	O_{3c}
**30**	557	1.26	0.67	553	O_{3c}
**31**	561	1.23	0.43	558	O_{3c}
**32**	559	1.29	0.41	555	O_{3c}
**33**	568	1.23	0.34	565	O_{3c}
**34**	565	1.21	0.29	562	O_{3c}
**35**	560	1.30	0.46	556	O_{3c}
**36**	538	1.73	0.67	531	O_{3c}
**37**	575	1.20	0.58	572	O_{3c}
**38**	544	1.63	0.58	538	O_{3c}
**39**	606	0.71	0.57	605	O_{3c}
**40**	550	1.69	0.86	543	Surface O_{3c}
**41**	557	1.42	0.49	553	Surface O_{3c}
**42**	552	1.13	0.69	549	Surface O_{3c}

**43**	527	1.50	0.73	522	Surface O_{3c}
**44**	202	6.22	0.53	116	OH
**45**	734	0.55	0.39	733	Surface O_{2c}
**46**	333	6.28	0.11	252	OH

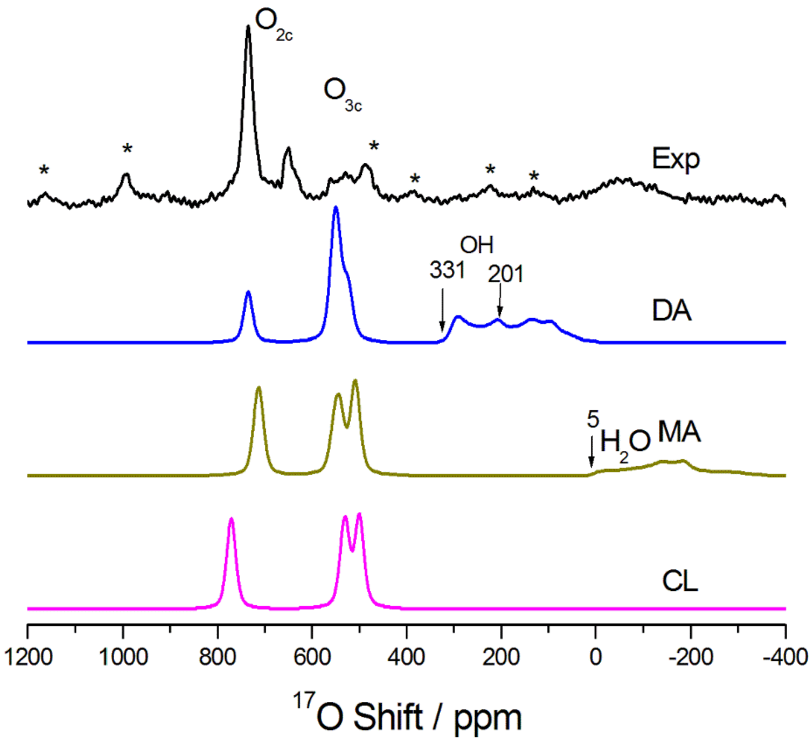

Supplementary Figure 24. Comparison of the experimental ^{17}O NMR spectrum of NO101-TiO_2 with the simulated spectra based on DFT calculation results using defect-free surface structures. NO101-TiO_2 (Exp) was surface selectively ^{17}O-labeled and vacuum-dried for 12 h. Simulated spectra are based on DFT calculation results using clean anatase TiO_2(101) (CL), hydrated anatase TiO_2(101) with $^1/_2$ ML water molecularly adsorbed (MA) and hydrated anatase TiO_2(101) $^1/_2$ ML water dissociatively adsorbed (DA), respectively. Isotropic chemical shifts of the three signals that have large C_Qs (> 6 MHz) have been marked.

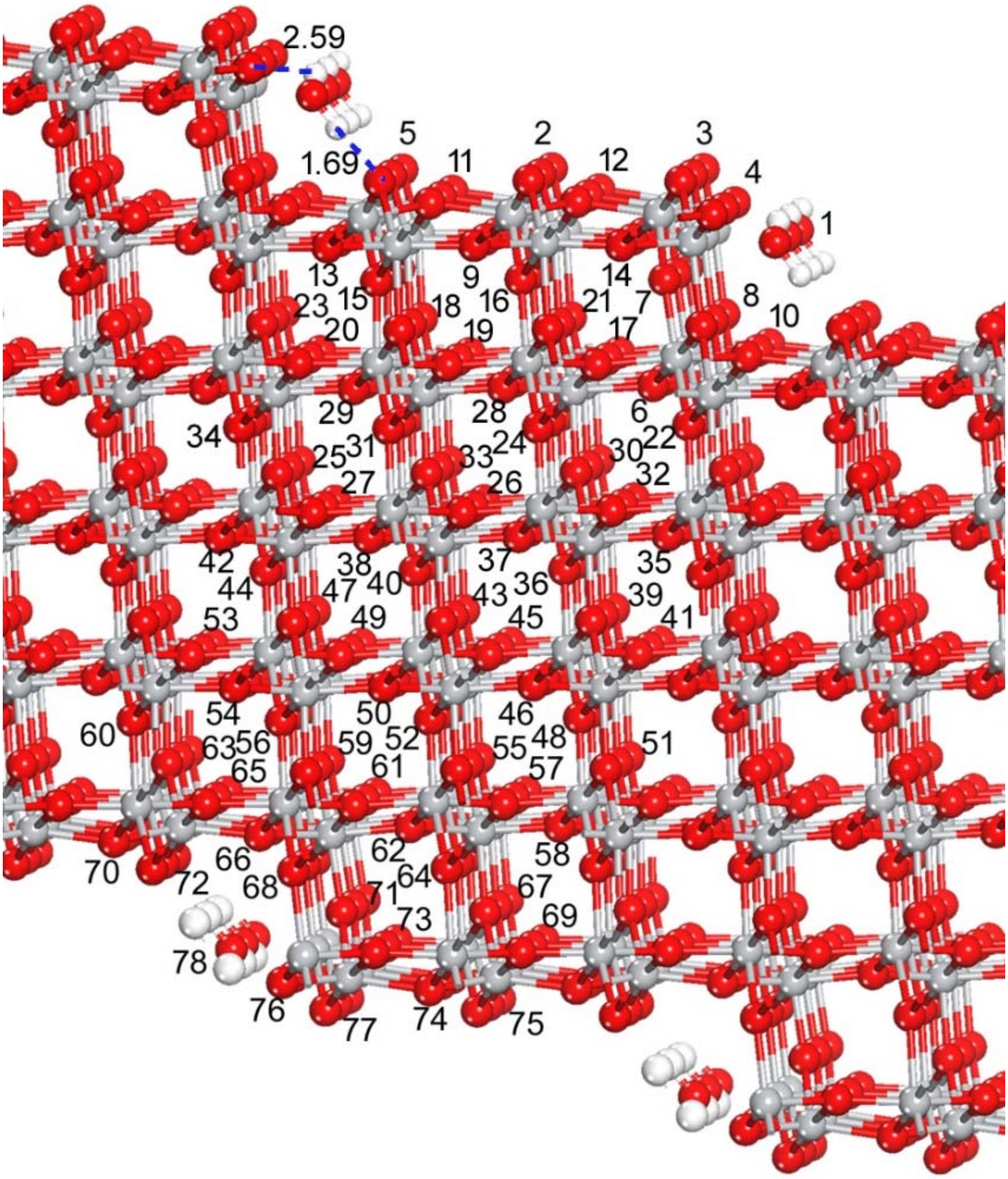

Supplementary Figure 25. Calculated structure of anatase TiO_2(134) vicinal surface with water molecularly adsorbed at step-edge Ti_{5c} in orientation A. The TiO_2(134) vicinal surface consists of type D steps and (101) planes. The adsorption orientation is denoted as OA in the text. Different oxygen sites are numbered, and their isotropic chemical shifts and quadrupolar parameters obtained with the DFT calculations are shown in Supplementary Table 13. δ_{ref} = 51.

Supplementary Table 13. Calculated NMR parameters for oxygen species in anatase TiO_2(134) vicinal surface with water molecularly adsorbed at step-edge Ti_{5c} in orientation A. Isotropic chemical shifts (δ_{iso}), quadrupolar parameters (C_Q and η) and center of gravity (δ_{CG}) of the NMR signals are listed below. δ_{ref} =51. Corresponding structure is described in Supplementary Fig. 25.

	δ_{iso}/ppm	C_Q/MHz	η	δ_{CG}/ppm	Assignment
**1**	21	8.37	0.71	-146	Water molecules absorbed at Ti_{5c} of the step edge
**2**	761	1.27	0.71	757	O_{2c} at flat terrace
**3**	730	1.09	1.00	727	O_{2c} at step edge
**4**	705	0.59	0.81	704	Another O_{2c} site at the step edge, which has a weak hydrogen bond with the adsorbed water molecule
**5**	650	1.74	0.24	644	O_{2c} at the flat terrace next to the step edge, which has a strong hydrogen bond with the adsorbed water molecule
**6**	558	1.28	0.55	554	Subsurface O_{3c}
**7**	552	1.33	0.95	547	Subsurface O_{3c}
**8**	547	1.60	0.13	542	Surface O_{3c}
**9**	536	1.38	0.81	531	Subsurface O_{3c}
**10**	520	1.39	0.57	516	Surface O_{3c}
**11**	499	1.12	0.97	496	Surface O_{3c}
**12**	488	1.21	0.93	484	Surface O_{3c}
**13**	537	1.48	0.68	532	Subsurface O_{3c}
**14**	548	1.31	0.87	544	Subsurface O_{3c}
**15**	549	1.23	0.63	546	O_{3c}
**16**	552	1.13	0.96	549	O_{3c}
**17**	554	1.37	0.56	550	O_{3c}
**18**	553	1.85	0.39	546	O_{3c}
**19**	552	1.27	0.47	548	O_{3c}
**20**	552	1.23	0.39	549	O_{3c}
**21**	567	1.72	0.29	561	O_{3c}
**22**	562	1.16	0.42	559	O_{3c}
**23**	565	1.39	0.51	561	O_{3c}
**24**	564	1.05	0.84	561	O_{3c}
**25**	563	1.34	0.46	559	O_{3c}
**26**	559	1.23	0.43	556	O_{3c}
**27**	560	1.23	0.38	557	O_{3c}
**28**	559	1.22	0.67	556	O_{3c}
**29**	562	1.25	0.50	559	O_{3c}
**30**	559	1.41	0.27	555	O_{3c}
**31**	561	1.05	0.75	558	O_{3c}
**32**	560	1.26	0.33	557	O_{3c}
**33**	559	1.44	0.32	555	O_{3c}
**34**	562	1.29	0.48	558	O_{3c}
**35**	561	1.22	0.40	558	O_{3c}
**36**	562	1.24	0.43	559	O_{3c}
**37**	560	1.24	0.40	557	O_{3c}

**38**	562	1.26	0.33	559	O_{3c}
**39**	562	1.23	0.47	559	O_{3c}

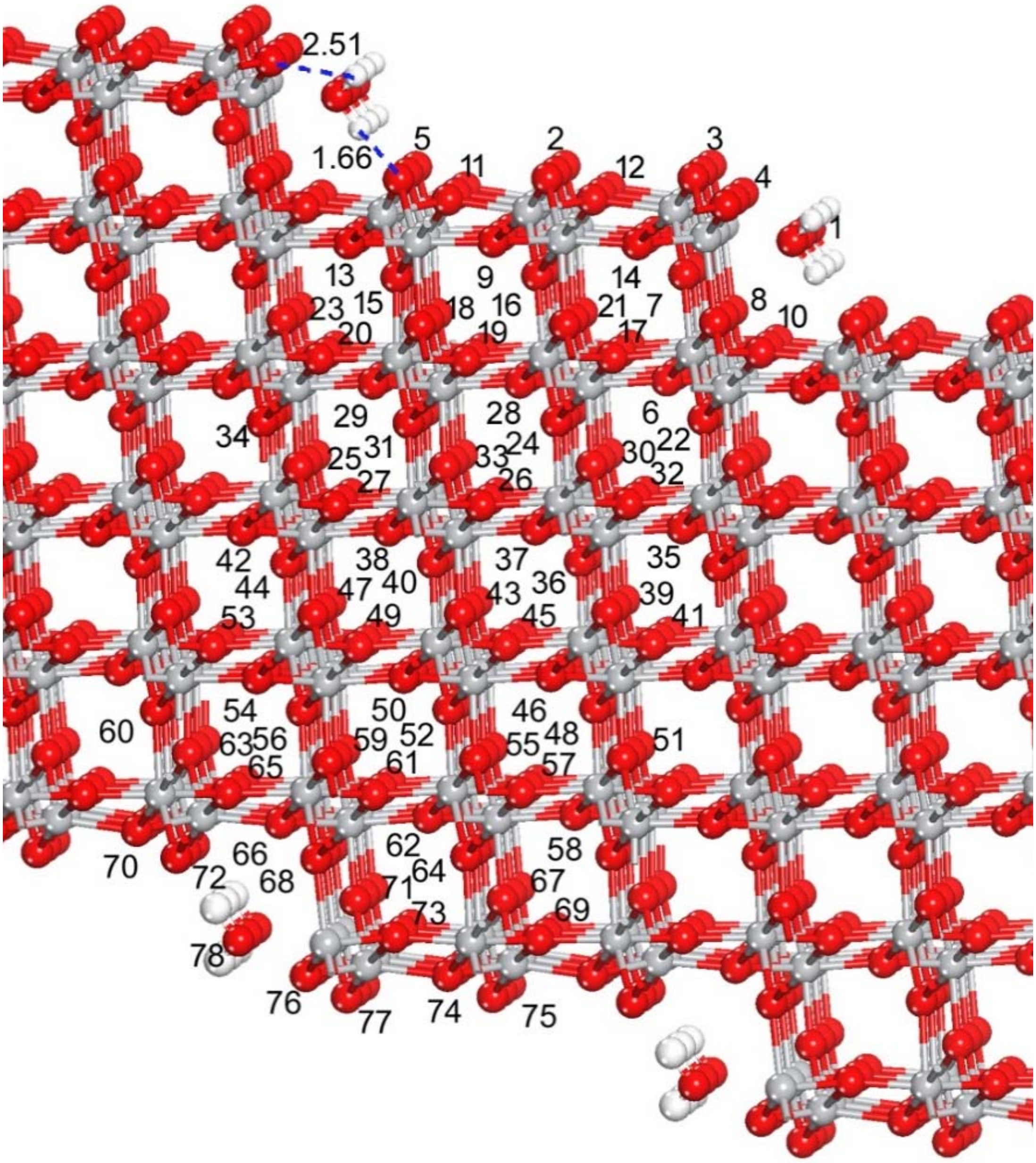

Supplementary Figure 26. Calculated structure of anatase TiO_2(134) vicinal surface with water molecularly adsorbed at step-edge Ti_{5c} in orientation B. The TiO_2(134) vicinal surface consists of type D steps and (101) planes. The adsorption orientation is denoted as OB in the text. Different oxygen sites are numbered, and their isotropic chemical shifts and quadrupolar parameters obtained with the DFT calculations are shown in Supplementary Table 14. δ_{ref} = 50.

Supplementary Table 14. Calculated NMR parameters for oxygen species in anatase TiO_2(134) vicinal surface with water molecularly adsorbed at step-edge Ti_{5c} in orientation B. Isotropic chemical shifts (δ_{iso}), quadrupolar parameters (C_Q and η) and center of gravity (δ_{CG}) of the NMR signals are listed below. δ_{ref} =50. Corresponding structure is described in Supplementary Fig. 26.

	δ_{iso}/ppm	C_Q/MHz	η	δ_{CG}/ppm	Assignment
**1**	7	8.58	0.70	-168	Water absorbed at Ti_{5c} of the step edge
**2**	756	1.27	0.74	752	O_{2c} at flat terrace
**3**	736	1.13	0.93	733	O_{2c} at step edge
**4**	718	0.80	0.41	717	Another O_{2c} site at the step edge, which has a weak hydrogen bond with the adsorbed water molecule
**5**	639	1.68	0.23	633	O_{2c} at the flat terrace next to the step edge, which has a strong hydrogen bond with the adsorbed water molecule
**6**	556	1.29	0.52	552	Subsurface O_{3c}
**7**	541	1.29	0.83	537	Subsurface O_{3c}
**8**	559	1.54	0.10	554	Surface O_{3c}
**9**	535	1.38	0.74	530	Subsurface O_{3c}
**10**	519	1.42	0.51	515	Surface O_{3c}
**11**	496	1.13	0.92	493	Surface O_{3c}
**12**	490	1.24	0.84	486	Surface O_{3c}
**13**	537	1.44	0.67	532	Subsurface O_{3c}
**14**	551	1.49	0.80	546	Subsurface O_{3c}
**15**	548	1.26	0.60	544	O_{3c}
**16**	552	1.13	0.96	549	O_{3c}
**17**	549	1.32	0.50	545	O_{3c}
**18**	551	1.85	0.40	544	O_{3c}
**19**	551	1.27	0.45	547	O_{3c}
**20**	551	1.24	0.39	548	O_{3c}
**21**	564	1.74	0.28	558	O_{3c}
**22**	560	1.12	0.46	557	O_{3c}
**23**	563	1.37	0.46	559	O_{3c}
**24**	563	1.05	0.80	560	O_{3c}
**25**	563	1.33	0.45	559	O_{3c}
**26**	558	1.23	0.41	555	O_{3c}
**27**	560	1.23	0.37	557	O_{3c}
**28**	560	1.24	0.65	556	O_{3c}
**29**	561	1.25	0.48	558	O_{3c}
**30**	559	1.42	0.32	555	O_{3c}
**31**	560	1.08	0.74	557	O_{3c}
**32**	560	1.26	0.31	557	O_{3c}
**33**	559	1.45	0.33	555	O_{3c}
**34**	562	1.29	0.48	558	O_{3c}
**35**	560	1.23	0.39	557	O_{3c}
**36**	562	1.26	0.44	559	O_{3c}
**37**	560	1.23	0.39	557	O_{3c}

**38**	561	1.26	0.32	558	O_{3c}
**39**	562	1.25	0.46	559	O_{3c}

Supplementary Table 15. Adsorption energies of water at TiO_2(101) and (134).

Model	Adsorption energy (eV)
Hydrated TiO_2(101) at a water coverage of $^1/_2$ ML (molecular adsorption)*	0.75
Hydrated TiO_2(101) at a water coverage of $^1/_2$ ML (dissociative adsorption)*	0.47
TiO_2(134) vicinal surface, water molecularly adsorbed at the step-edge Ti_{5c} in orientation A (OA)**	1.01
TiO_2(134) vicinal surface, water molecularly adsorbed at the step-edge Ti_{5c} in orientation B (OB)**	0.98

* $^1/_2$ ML means each two surface Ti_{5c} adsorb one water molecule.

** Each two step-edge Ti_{5c} adsorb one water molecule.

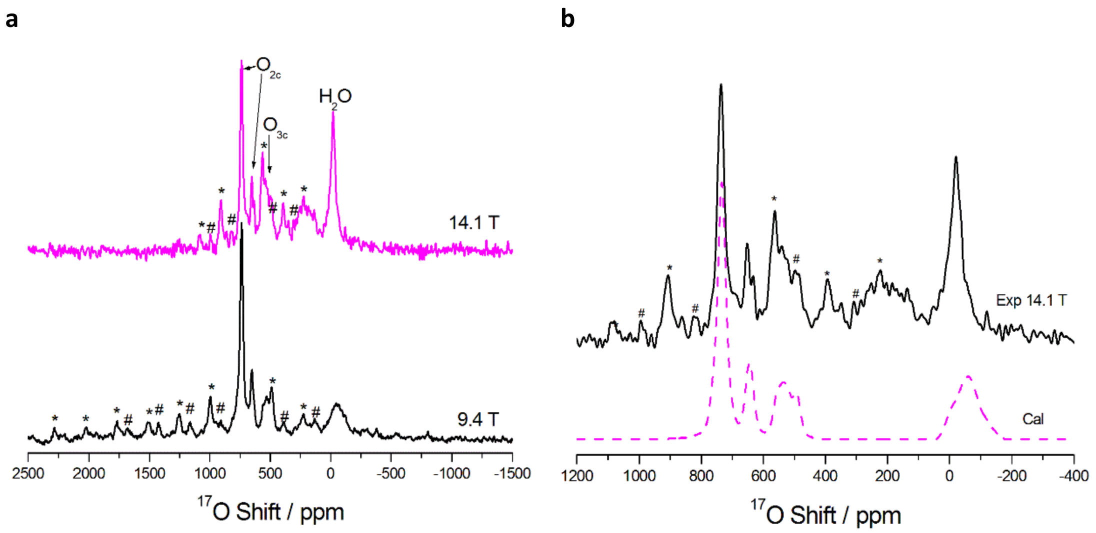

Supplementary Figure 27. ^{17}O NMR spectra of NO101-TiO_2 obtained at different external magnetic field strengths. a, Comparison of the ^{17}O spin-echo NMR spectra of fully dried NO101-TiO_2 obtained by NMR spectrometers of 9.4 T (bottom) and 14.1 T (top), respectively. A rotor synchronized Hahn-echo sequence ($\pi/6$ - τ - $\pi/3$ - τ - acquisition) and an optimized recycle delay (0.5 s), with ^1H decoupling, were used to obtain the NMR data. The sample was packed into a 4 mm zirconia rotor, and the NMR spectra were obtained at a spinning rate of 14 kHz. 110000 (9.4 T) and 104000 (14.1 T) scans were acquired, respectively. **b**, The ^{17}O spin-echo spectrum of the fully dried ^{17}O-labeled NO101-TiO_2 obtained at 14.1 T (solid black line) in comparison to the simulation (dashed line) according to the DFT calculation results using the model of anatase TiO_2(134) vicinal surface consisting of type D steps and (101) planes, which adsorbs water molecules in two orientations. The proportion of the peak area was set the same as in Fig. 3, which is shown in Supplementary Table 16. Asterisks denote sidebands of the O_{2c} signal centered at 730 ppm, and #s denote sidebands of the O_{2c} signal centered at 640 ppm.

Supplementary Table 16. Parameters used for simulating the ^{17}O NMR spectra of the 12 h-vacuum-dried NO101-TiO_2 in Fig. 3 and Supplementary Fig. 27b. These parameters contain the isotropic chemical shifts (δ_{iso}), quardropolar parameters (C_Q and η), center of gravity (δ_{CG}) of the NMR signals and the percentage.

	OA					OB				
**O Site#**	**δ_{iso} / ppm**	**C_Q / MHz**	**η**	**δ_{CG} / ppm**	**Percent/ %***	**δ_{iso} / ppm**	**C_Q / MHz**	**η**	**δ_{CG} / ppm**	**Percent /%***
**1**	21	8.37	0.71	-143	13.5	7	8.58	0.70	-165	13.5
**2**	761	1.27	0.71	760	2.5	756	1.27	0.74	755	2.5
**3**	730	1.09	1.00	730	16.8	736	1.13	0.93	736	16.8
**4**	705	0.59	0.81	707	1.8	718	0.80	0.41	720	1.8
**5**	650	1.74	0.24	647	5.6	639	1.68	0.23	636	5.6
**6**	558	1.28	0.55	557	1.7	556	1.29	0.52	555	1.7
**7**	552	1.33	0.95	550	0.3	541	1.29	0.83	540	0.3
**8**	547	1.60	0.13	545	0.3	559	1.54	0.10	557	0.3
**9**	536	1.38	0.81	534	1.6	535	1.38	0.74	533	1.6
**10**	520	1.39	0.57	519	2.0	519	1.42	0.51	518	2.0
**11**	499	1.12	0.97	499	1.0	496	1.13	0.92	496	1.0
**12**	488	1.21	0.93	487	2.0	490	1.24	0.84	489	2.0
**13**	537	1.48	0.68	532	0.4	537	1.44	0.67	532	0.4
**14**	548	1.31	0.87	544	0.3	551	1.49	0.80	546	0.3
**Total**	/	/	/	/	50.0	/	/	/	/	50.0

Oxygen site are numbered according to Fig. 3 in the manuscript.

*These parameters are given by the simulation, and other parameters are from the DFT calculations. The contribution of each oxygen species in sideband signals has not been considered.

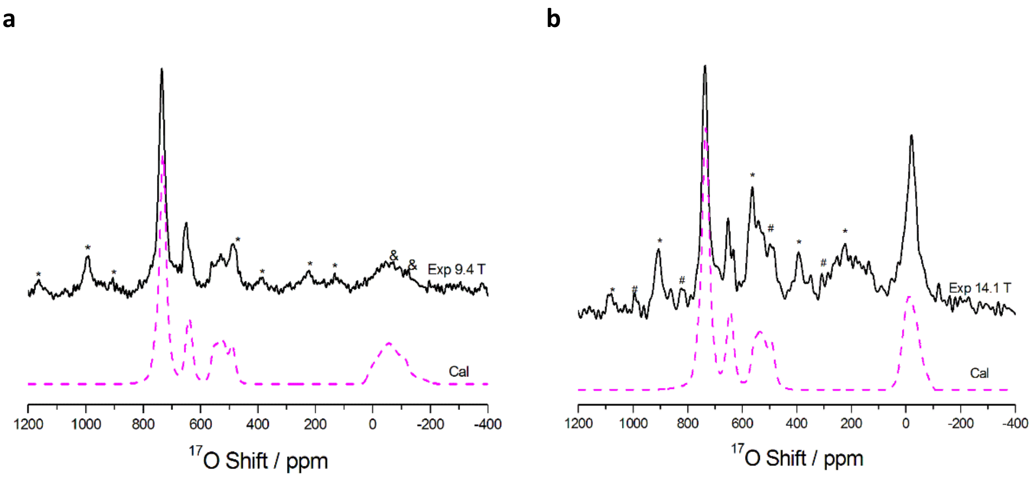

Supplementary Figure 28. The ^{17}O NMR spectra of NO101-TiO$_2$ obtained at different external magnetic field strengths in comparison to the simulated spectra. **a**, 9.4 T and **b,** 14.1 T. NO101-TiO$_2$ had been ^{17}O-labeled and fully dried. Solid black lines are experimental spectra, and dashed magenta lines represent simulated ones. In addition to the percentage of the peak area of each adopted oxygen sites, C_Qs of the adsorbed water in both orientations were allowed to be adjustable in the simulation to achieve better fitting. Other parameters used in the simulation are given by DFT calculations for the model of anatase TiO$_2$(134) vicinal surface consisting of type D steps and (101) planes, which adsorbs water molecules in two orientations.

Supplementary Note 6

The C_Qs of the adsorbed water used to fit the spectra in Supplementary Fig. 28 are 5.40 MHz for OA and 6.50 MHz for OB, respectively, which are significantly smaller than those given by the DFT calculation (8.37 and 8.58 MHz, respectively, Supplementary Tables 13-14). This may be attributed to the motion of the adsorbed water, which is similar to the observed ^{2}H static NMR signals from rigid and mobile water.[10]

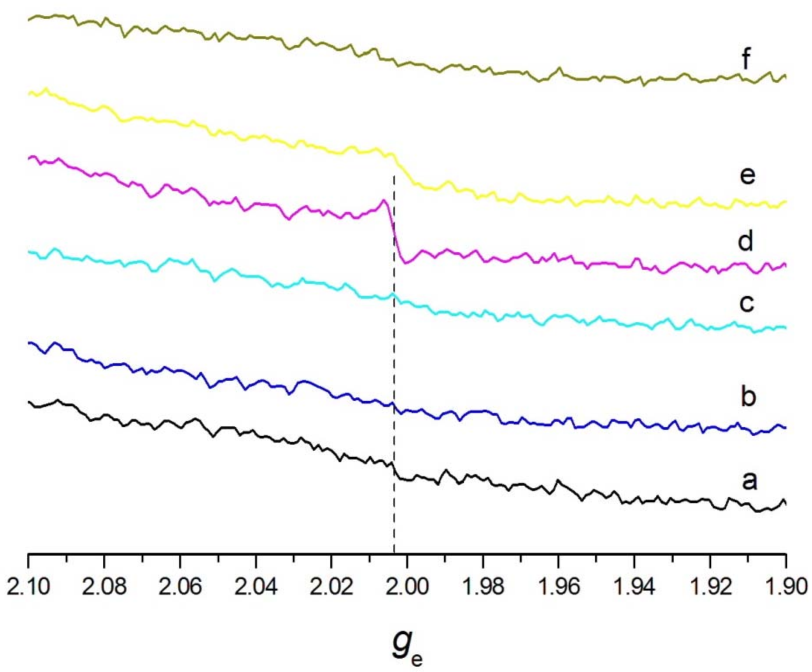

Supplementary Figure 29. Room-temperature electron paramagnetic resonance spectra of the two faceted nanocrystals. a~c: NS001-TiO_2, **a**, vacuum dried at 100 °C for 1.5 h, **b**, vacuum dried at room temperature for 12 h and **c**, saturated with water vapor. **d~f**: NO101-TiO_2, **d**, vacuum dried at 100 °C for 1.5 h, **e**, vacuum dried at room temperature for 12 h and **f**, saturated with water vapor. The resonance centered at 2.003 is the evidence of oxygen vacancies[11].

Supplementary Note 7

The dissociation of $H_2{}^{17}O$ on the oxygen vacancies generated in the vacuum-drying pretreatment at 100 °C and subsequent migration of oxygen ions are possibly the origin of the surface ^{17}O NMR signals of NO101-TiO_2. It should be mentioned that, the NO101-TiO_2 sample was ^{17}O-labeled with excess $H_2{}^{17}O$ (Supplementary Fig. 10, bottom line). Tilocca *et al*. have pointed out that hydrogen bonds between water molecules reduce the dissociation barrier of water on (101) facets of anatase TiO_2, therefore, water molecules incorporated in a monolayer or a bilayer over a surface with oxygen vacancies dissociate spontaneously at 160 K[12]. On the other hand, after most of the adsorbed water was removed by vacuum-drying, the hydroxyl groups on NO101-TiO_2 are likely to recombine to form water molecules, which were expected to undergo molecular adsorption instead of dissociation adsorption. These are probably the reason why hydroxyl group was barely observed on the vacuum-dried NO101-TiO_2 sample[13].

Supplementary Reference

1. Noberi, C., Zaman, A. C., Ustundag, C. B., Kaya, F., Kaya, C. Electrophoretic deposition of hydrothermally synthesised Ag-TiO2 hybrid nanoparticles onto 3-D Ni filters. *Mater. Lett.* **67**, 113-116 (2012).
2. Yang, H. G., Sun, C. H., Qiao, S. Z., Zou, J., Liu, G., Smith, S. C., Cheng, H. M. Lu, G. Q. Anatase TiO_2 single crystals with a large percentage of reactive facets. *Nature* **453**, 638-U634 (2008).
3. Liu, L. C., Gu, X. R., Ji, Z. Y., Zou, W. X., Tang, C. J., Gao, F., Dong, L. Anion-assisted synthesis of TiO_2 nanocrystals with tunable crystal forms and crystal facets and their photocatalytic redox activities in organic reactions. *J. Phys. Chem. C* **117**, 18578-18587 (2013).
4. Wang, M., Wu, X. P., Zheng, S. J., Zhao, L., Li, L., Shen, L., Gao, Y. X., Xue, N. H., Guo, X. F., Huang, W. X., Gan, Z. H., Blanc, F., Yu, Z. W., Ke, X. K., Ding, W. P., Gong, X. Q., Grey, C. P., Peng, L. M. Identification of different oxygen species in oxide nanostructures with ^{17}O solid-state NMR spectroscopy. *Sci. Adv.* **1**, e1400133 (2015).
5. Ye, L. Q., Mao, J., Liu, J. Y., Jiang, Z., Peng, T. Y., Zan, L. Synthesis of anatase TiO2 nanocrystals with {101}, {001} or {010} single facets of 90% level exposure and liquid-phase photocatalytic reduction and oxidation activity orders. *J. Mater. Chem. A* **1**, 10532-10537 (2013).
6. Zhao, L., Qi, Z., Blanc, F., Yu, G. Y., Wang, M., Xue, N. H., Ke, X. K., Guo, X. F., Ding, W. P., Grey, C. P., Peng, L. M. Investigating local structure in layered double hydroxides with ^{17}O NMR spectroscopy. *Adv. Funct. Mater.* **24**, 1696-1702 (2014).
7. Peng, L. M., Liu, Y., Kim, N. J., Readman, J. E., Grey, C. P. Detection of Bronsted acid sites in zeolite HY with high-field ^{17}O MAS NMR techniques. *Nature Mater.* **4**, 216-219 (2005).
8. Peng, L. M., Huo, H., Liu, Y. and Grey, C. P. O-17 magic angle spinning NMR studies of brønsted acid sites in zeolites HY and HZSM-5. *J. Am. Chem. Soc.* **129**, 335-346 (2007).
9. Lippmaa, E., Samoson, A. and Mägi, M. High-resolution aluminum-27 NMR of aluminosilicates. *J. Am. Chem. Soc.*, **108**, 1730-1735 (1986).
10. Li, S. H., Zheng, A. M., Su, Y. C., Fang, H. J., Shen, W. L., Yu, Z. W., Chen, L. Deng, F. Extra-framework aluminium species in hydrated faujasite zeolite as investigated by two-dimensional solid-state NMR spectroscopy and theoretical calculations. *Phys. Chem. Chem. Phys.*, **12**, 3895–3903 (2010).
11. Liu, H., Ma, H. T., Li, X. Z., Li, W. Z., Wu, M., Bao, X. H. The enhancement of TiO_2 photocatalytic activity by hydrogen thermal treatment. *Chemosphere* **50**, 39-46 (2003).
12. Tilocca, A., Selloni, A. Structure and reactivity of water layers on defect-free and defective anatase TiO_2(101) surfaces. *J. Phys. Chem. B* **108**, 4743-4751(2004).
13. Stirling, A., Bernasconi, M., Parrinello, M. Ab initio simulation of water interaction with the (100) surface of pyrite. *J. Chem. Phys.* 118, 8917-8926 (2003).
